# Evaluation of Supercritical CO_2_-Assisted Protocols in a Model of Ovine Aortic Root Decellularization

**DOI:** 10.3390/molecules25173923

**Published:** 2020-08-27

**Authors:** Elvira R. Gafarova, Ekaterina A. Grebenik, Alexey E. Lazhko, Anastasia A. Frolova, Anastasia S. Kuryanova, Alexandr V. Kurkov, Ilya A. Bazhanov, Byron S. Kapomba, Nastasia V. Kosheleva, Ivan A. Novikov, Anatoly B. Shekhter, Elena N. Golubeva, Anna B. Soloviova, Peter S. Timashev

**Affiliations:** 1 Institute for Regenerative Medicine, Sechenov First Moscow State Medical University, 119991 Moscow, Russia; grebeneka@gmail.com (E.A.G.); nastyfr@ya.ru (A.A.F.); stassy2202@gmail.com (A.S.K.); a-kurkov@yandex.ru (A.V.K.); ilyabazhanov@yandex.ru (I.A.B.); byronkaps@gmail.com (B.S.K.); a.shehter@yandex.ru (A.B.S.); timashev.peter@gmail.com (P.S.T.); 2 Kurnakov Institute of General and Inorganic Chemistry, Russian Academy of Sciences, 119991 Moscow, Russia; alexeylazhko@mail.ru; 3 N. N. Semenov Federal Research Center for Chemical Physics, Russian Academy of Sciences, 117977 Moscow, Russia; ann.solovieva@gmail.com; 4 FSBSI “Institute of General Pathology and Pathophysiology”, 125315 Moscow, Russia; n_kosheIeva@mail.ru; 5 Faculty of Biology, Lomonosov Moscow State University, 119234 Moscow, Russia; 6 Scientific Research Institute of Eye Diseases, 119021 Moscow, Russia; i.novikov@niigb.ru; 7 Faculty of Chemistry, Lomonosov Moscow State University, 119991 Moscow, Russia; legol@mail.ru

**Keywords:** biomaterials, aortic valve, decellularization, supercritical CO_2_, biomedical engineering

## Abstract

One of the leading trends in the modern tissue engineering is the development of new effective methods of decellularization aimed at the removal of cellular components from a donor tissue, reducing its immunogenicity and the risk of rejection. Supercritical CO_2_ (scCO_2_)-assisted processing has been proposed to improve the outcome of decellularization, reduce contamination and time costs. The resulting products can serve as personalized tools for tissue-engineering therapy of various somatic pathologies. However, the decellularization of heterogeneous 3D structures, such as the aortic root, requires optimization of the parameters, including preconditioning medium composition, the type of co-solvent, values of pressure and temperature inside the scCO_2_ reactor, etc. In our work, using an ovine aortic root model, we performed a comparative analysis of the effectiveness of decellularization approaches based on various combinations of these parameters. The protocols were based on the combinations of treatments in alkaline, ethanol or detergent solutions with scCO_2_-assisted processing at different modes. Histological analysis demonstrated favorable effects of the preconditioning in a detergent solution. Following processing in scCO_2_ medium provided a high decellularization degree, reduced cytotoxicity, and increased ultimate tensile strength and Young’s modulus of the aortic valve leaflets, while the integrity of the extracellular matrix was preserved.

## 1. Introduction

Aortic valve disorder is a widespread condition often requiring surgical replacement of the valve [1]. However, existing prostheses possess certain drawbacks, which limit their distribution in the clinical practice [2,3]. For example, the implantation of a mechanical prosthesis requires a life-long anticoagulant therapy associated with the risks of anticoagulant-related hemorrhage [4,5]. An alternative approach is based on the application of biological prostheses taking favor of superior hemodynamic properties and the avoidance of a chronic anticoagulant therapy. Traditionally, such valves are made from animal tissues treated with crosslinking agents in order to reduce the antigenicity. As a result of this treatment, the implanted valve undergoes continuous calcification requiring repeated surgical intervention [6]. In recent decades, numerous methods for valve decellularization have been developed [7,8]. Decellularization is a method of processing biomaterial aimed at removing cells from tissue while preserving the extracellular matrix (ECM) composition and architectonics [9,10]. The use of this method for the fabrication of aortic valve prostheses is meant to reduce the immunogenicity of the grafts while preserving hemodynamic function and improving the long-term prognosis for patients undergoing valve replacement surgery [11,12].

Existing technologies for decellularization include treatment with detergents (sodium dodecyl sulfate, SDS [13], sodium deoxycholate, SD [14], Triton X-100 [15], etc.), enzymes (trypsin) [16,17] and alkali [18], as well as the methods of cyclic freezing—thawing and exposure to high pressures (up to 1 GPa) [19]. However, the use of detergents at their working concentrations (typically 1%) often increases the cytotoxicity of the graft and requires time-consuming washing (up to 72 h) with a risk of bacterial contamination [20]. Enzymatic hydrolysis and the method of cyclic freezing–thawing are less effective and disrupt the structural organization of ECM [17,21]. The method of decellularization under the exposure to a high pressure requires complex and expensive hardware.

Since 2008, research has begun in the field of the decellularization of mammalian tissues with scCO_2_ [22]. Relative chemical inertness, favorable economic characteristics, such as cheapness and facile accessibility, as well as relatively low values of critical pressure (7.38 MPa) and temperature (31.1 °C), make scCO_2_ a commercially viable solvent [23,24]. Due to the minimal environmental impact, the scCO_2_ is referred to as a solvent of the so-called “green chemistry” and is widely used in the food, textile, perfume and cosmetic industries [25,26]. 

The mechanisms underlying scCO_2_-assisted decellularization are debatable and may be dual. On the one hand, owing to its ability to solubilize non-polar molecules, scCO_2_ can act as a decellularizing agent removing lipids—the structural building blocks of cellular membranes, while preserving polar molecules such as proteins of the ECM [27]. On the other hand, the high pressure in the reactor can burst the cells inside the tissue followed by the removal of cellular fragments upon rapid depressurization. When employed in a combination with traditional decellularizing agents [28,29,30] or co-solvents such as ethanol [22,31,32], scCO_2_ assists the decellularization and reduces the risk of contamination and processing time.

Along with the decellularization, scCO_2_ is considered as a promising sterilizing agent for medical devices [33,34,35,36]. The combination of such stages as decellularization and sterilization in one environmentally friendly technological process can significantly reduce energy costs and increase economic efficiency in the production of transplants. All these advantages make scCO_2_ a potentially powerful tool for tissue decellularization. 

However, the decellularization of 3D heterogeneous tissue structures, such as the aortic root, in scCO_2_ medium requires optimization of the parameters, including preconditioning medium composition, type of co-solvent, values of pressure and temperature inside the scCO_2_ reactor, etc., in order to achieve complete cell removal while preserving the ECM structure. In our work, using an ovine aortic root model, we performed a comparative analysis of the effectiveness of decellularization using approaches based on various combinations of these parameters. The protocols were based on combinations of treatment in alkaline, ethanol or detergent solutions with scCO_2_-assisted processing at different modes.

## 2. Results

**Figure 1:**
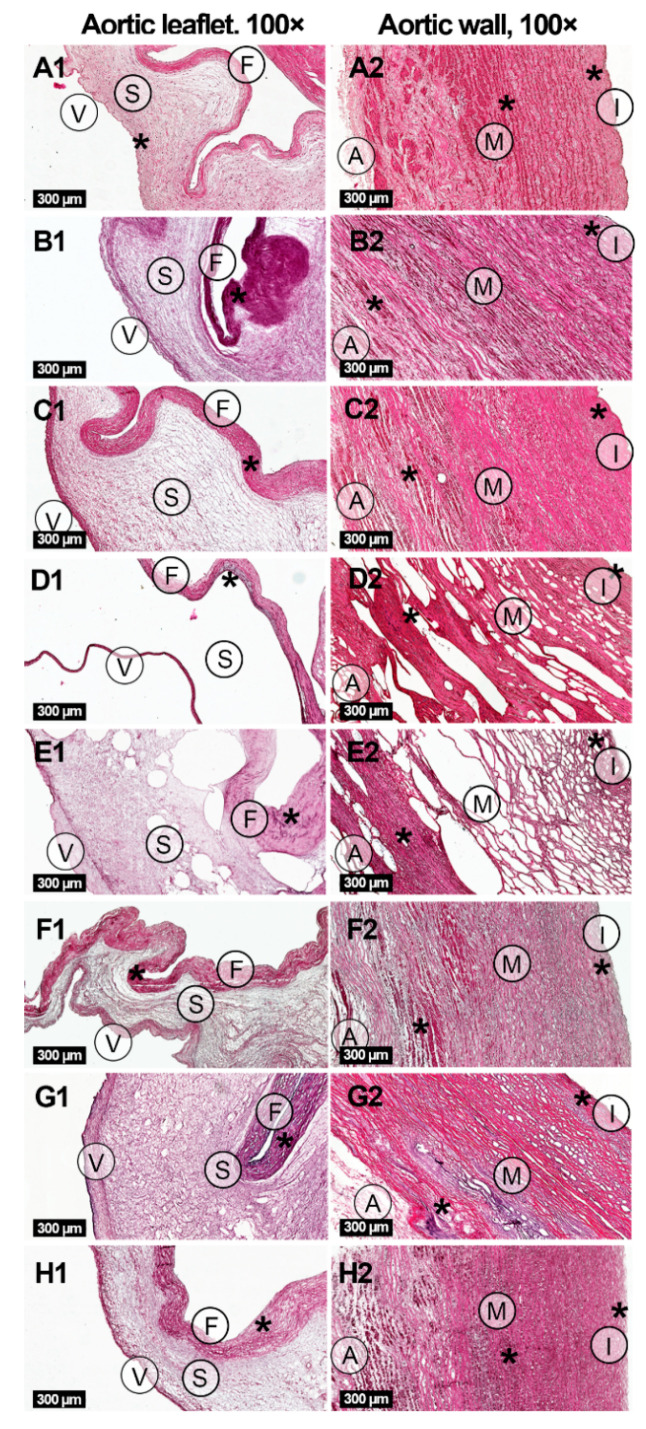
Histological study of native (**A1**,**A2**) and decellularized ovine aortic roots under hematoxylin-eosin staining. Decellularization protocols included scCO_2_-assisted processing at the pressure of 15 MPa (**B1**,**B2**) and 25 MPa (**C1**,**C2**) with the use of ethanol as a co-solvent; combinations of alkaline treatment with scCO_2_-assisted processing at the pressure of 10 MPa (**D1, D2**) and 15 MPa (**E1**,**E2**); detergents alone (**F1**,**F2**) or with following scCO_2_ treatment at the pressure of 15 MPa (**G1**,**G2**) and 25 MPa (**H1**,**H2**). Three layers are indicated in aortic valve leaflets: ventricularis (V), fibrosa (F) and spongiosa (S). Additionally, three layers are indicated in aortic walls: intima (I), media (M) and adventitia (A). Typical accumulations of cells are shown with asterisks (*, see Supplementary Figure S1 for enlarged images).

The histological analysis of the ovine aortic roots indicated three layers in aortic valve leaflets of all studied groups: ventricularis, fibrosa and spongiosa (Figure 1, Supplementary Figure S1). The aortic walls in all groups were also composed of three layers: intima, media and adventitia.

**Figure 2:**
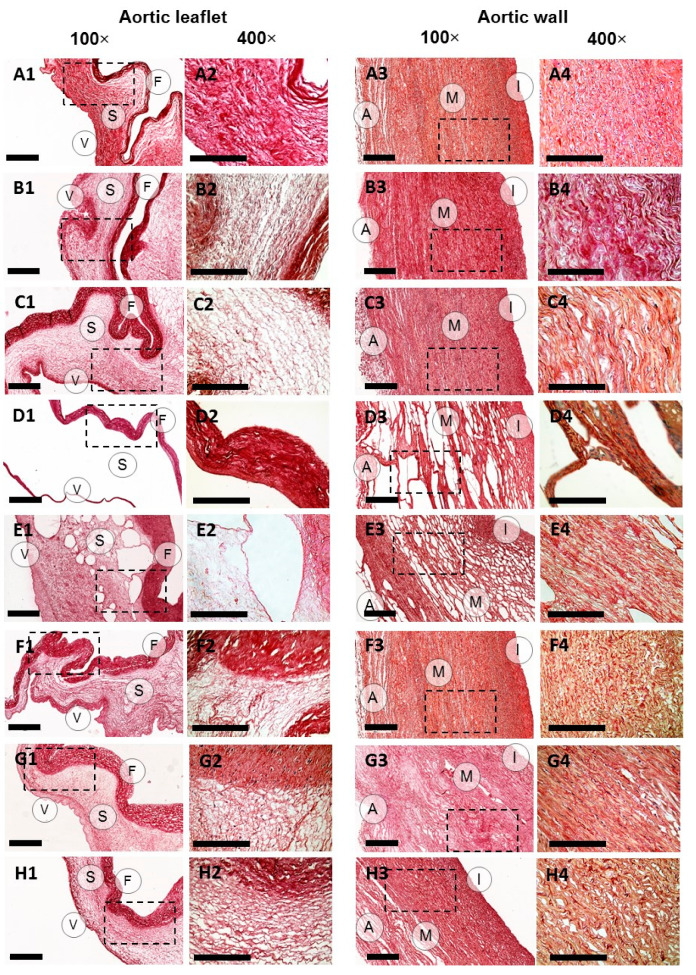
Collagen content in aortic valve leaflets and walls of native (**A1–4**) and decellularized ovine aortic roots. Decellularization protocols included scCO_2_-assisted processing at the pressure of 15 MPa (**B1–4**) and 25 MPa (**C1–4**) with the use of ethanol as a co-solvent; combinations of alkaline treatment with scCO_2_-assisted processing at the pressure of 10 MPa (**D1–4**) and 15 MPa (**E1–4**); detergents alone (**F1–4**) or with following scCO_2_ treatment at the pressure of 15 MPa (**G1–4**) and 25 MPa (**H1–4**). The collagen fibers were stained red with Picrosirius red. Dashed lines mark the areas where the 400× images were acquired. 100× scale bar = 100 µm; 400× scale bar = 300 µm.

**Figure 3:**
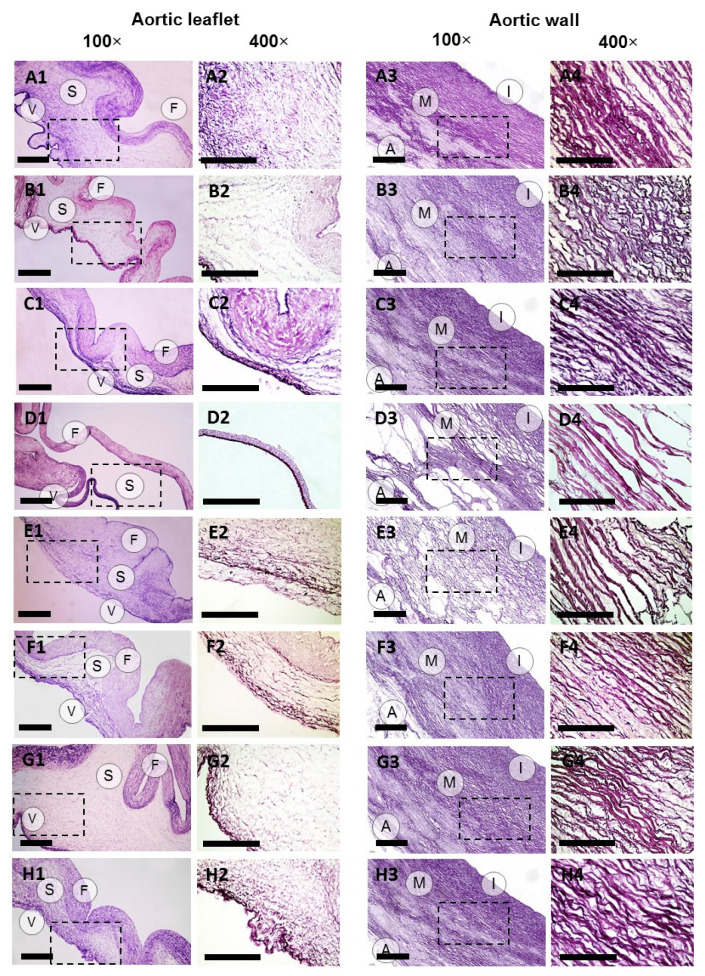
Elastin content in aortic valve leaflets and walls of native (**A1–4**) and decellularized ovine aortic roots. Decellularization protocols included scCO_2_-assisted processing at the pressure of 15 MPa (**B1–4**) and 25 MPa (**C1–4**) with the use of ethanol as a co-solvent; combinations of alkaline treatment with scCO_2_-assisted processing at the pressure of 10 MPa (**D1–4**) and 15 MPa (**E1–4**); detergents alone (**F1–4**) or with following scCO_2_ treatment at the pressure of 15 MPa (**G1–4**) and 25 MPa (**H1–4**). Weigert staining. Dashed lines mark the areas where the 400× images were acquired. 100× scale bar = 100 µm; 400× scale bar = 300 µm.

Picrosirius red staining applied with bright-field and polarized light microscopy techniques confirmed the preservation of collagen fibers in ECM in all sample groups (Figure 2, Supplementary Figure S2). However, significant structural differences were found between the groups, including the degree of changes in the sample histoarchitecture, decellularization and ECM organization (Figure 1, Figure 2 and Figure 3, Supplementary Figures S1–S8) causing alterations in mechanical properties (Table 1). In general, the processing in scCO_2_ medium increased ultimate tensile strength (UTS) and Young’s modulus of aortic valve leaflets, but to different extents depending on a protocol. Hematoxylin-eosin staining revealed retained focal accumulations of intact cells (without karyopyknosis, karyolysis, plasmolysis) in some experimental groups (Figure 1, Supplementary Figure S1).

### 2.1. Ethanol-Based Decellularization

The decellularization of ovine aortic roots by means of scCO_2_-assisted processing with the use of ethanol as a co-solvent under the pressure of either 15 or 25 MPa did not lead to either significant changes in host architecture, or to any significant degree of decellularization (Figure 1, Supplementary Figures S3 and S5). However, a marked redistribution of fibers in the ECM appeared (Figure 2 and Figure 3, Supplementary Figure S6). In particular, this protocol promoted condensation (increasing density) of collagen fibers in ventricularis. The density of ventricularis collagen fibers was significantly higher than that of the rest of the protocols, except for the alkali-based treatment. Macroscopic examination indicated a slight compressing of the aortic roots, presumably caused by dehydration (Supplementary Figure S9). This is also supported by the thinner valve leaflets as compared to the native sample (Table 1). Manually, the samples were stiffer than prior to the processing.

Condensation of the elastic fibers in the aortic walls occurred in the intima and was more pronounced at the pressure of 25 MPa when compared to 15 MPa. The density of elastic fibers was the greatest among all the protocols we studied. Elastic lamellae also condensed in the media and elastic fibers—in spongiosa of valve leaflets at 25 MPa and were denser than in most other experimental groups (Supplementary Figure S6). However, the differences were not statistically significant compared to that achieved at 15 MPa. Young’s moduli of the aortic walls increased correspondingly as well as UTS in the circumferential direction (Table 1). 

Interestingly, after scCO_2_-assisted processing at 15 MPa, the elastic fibers of fibrosa loosened due to the decellularization, while the increased pressure (25 MPa) condensed the fibers. However, the increase in Young’s modulus of the leaflets occurred in both cases (Table 1). The decrease in strain at fracture was less pronounced at the higher pressure most likely due to the tissue shrinkage.

As the result of the decellularization procedure, a slight decrease in the number of valve fibroblasts and aortic endotheliocytes was achieved independent of the pressure value. Additionally, at the pressure of 25 MPa, removal of smooth muscle cells occurred in the aortic walls (Figure 1, Supplementary Figure S5). 

The content of glycosaminoglycans (GAGs) decreased in the aortic walls with no significant difference at 15 and 25 MPa and with the native tissue (Supplementary Figures S4 and S7).

### 2.2. Alkali-Based Decellularization

In contrast to the previous protocol, decellularization in scCO_2_ at 15 MPa after preconditioning in an alkali-salt solution caused conspicuous structural changes in both the aortic wall and valve leaflet ECM (Figure 2 and Figure 3, Supplementary Figure S6) and an enormous increase in the Young’s modulus of the valve leaflets (Table 1). This meant that we performed additional series of experiments with the pressure value reduced to 10 MPa. However, all samples, regardless of the pressure value (10 or 15 MPa), acquired high porosity and were almost deprived of spongiosa, which significantly distinguished this protocol from all others. As expected, the thinner valve leaflets demonstrated higher UTS values as compared to the native tissue (Table 1).

Macroscopically, the tissues appeared slightly reddish and rough following scCO_2_ treatment (Supplementary Figure S9). Unlike with the previous ethanol-based protocols, the alkali-based decellularization provided the removal of a substantial number of smooth muscle cells from the aortic walls (Figure 1, Supplementary Figure S5). As per endotheliocytes and fibroblasts, the significance was higher when compared with the ethanol-based decellularization at 15 MPa (both in the aortic walls and valve leaflets) and 25 MPa (less endotheliocytes in valve leaflets and fibroblasts in the aortic walls).

ECM reorganization following alkali-based decellularization included condensation of collagen fibers in ventricularis of the valve leaflets, which was comparable to the ethanol-based protocol with the maximum at the pressure of 10 MPa. In the aortic walls, a loosening of the fibers of the media was detected, which is comparable with the other protocols. Similarly, Young’s modulus was the orders higher for the valve leaflets but lower for the aortic walls after the scCO_2_ treatment (Table 1). Even though the decellularization reduced the thickness of the leaflets, in the aortic walls, it was preserved, presumably due to the tissue shrinkage in the longitudinal direction, increasing the strain at fracture and porosity. 

The collagen fiber packing in the aortic walls and valve leaflets was affected differently depending on the pressure value. For example, at 10 MPa, the collagen fibers in spongiosa were more loosely arranged compared to that in the native tissue and after the ethanol-based decellularization, while at 15 MPa, collagen fiber packing in fibrosa was denser (Figure 2, Supplementary Figures S2 and S6).

The elastic fibers in fibrosa and elastic lamellae in media appeared loosened after alkali-based decellularization at both 10 and 15 MPa. The loosening of the elastic fibers in fibrosa at the pressure of 15 MPa was less pronounced than after the ethanol-based decellularization at 15 MPa, but higher than in all other groups. Loosening of the elastic lamellae in media at both 10 and 15 MPa also significantly differed from other protocols except for the detergent treatment followed by scCO_2_-assisted processing at the pressure of 25 MPa. The arrangement of elastic fibers in the intima was similar to that after the ethanol-based decellularization. Loosening of the elastic fibers in ventricularis took place only at 15 MPa (Figure 3, Supplementary Figure S6). 

As opposed to the ethanol-based decellularization, the use of alkali led to a significant decrease in the content of GAGs in the aortic walls and valve leaflets independent of the pressure value (Supplementary Figures S4 and S7).

### 2.3. Detergent-Based Decellularization

The state-of-the-art protocol based on treatment in a detergent solution (SDS/SD) provided relatively high level of decellularization including removal of endotheliocytes and fibroblasts from the aortic walls and valve leaflets, as well as smooth muscle cells from the aortic walls, with slight alterations in the ECM and histoarchitecture (Figure 1, Figure 2 and Figure 3, Supplementary Figures S2–S8). Following decellularization, the aortic roots maintained their gross appearance, but whitened. The extent of the decellularization of the aortic valve leaflets, as well as the removal of fibroblasts and smooth muscle cells from the aortic walls was higher than that of ethanol-based protocols and comparable with the alkaline treatment. However, the number of endotheliocytes in aortic walls did not differ from all previously described methods (Supplementary Figure S5). The inclusions of residual cellular material (fragments of cell membranes and nuclei) were identified in dense and deep structures, mostly between collagen fiber bundles in the pars fibrosa and spongiosa of the valve leaflets and aortic media. 

After the decellularization, the collagen fiber packing was only affected in the aortic valve leaflet fibrosa and spongiosa, leading to their looser arrangement. For the fibrosa, this effect was even more significant than after the ethanol- and alkali-based treatments (Figure 2, Supplementary Figure S6).

In contrast, the distribution of elastic fibers in the spongiosa was the most widespread among all the experimental groups (Figure 3, Supplementary Figure S6) indicating a different sensitivity of elastin and collagen to the detergent treatment.

With this treatment protocol, the content of GAGs in the valve leaflets and aortic walls was lower than that after the ethanol-based decellularization. However, in the aortic walls, it was higher when compared to that after the alkaline treatment (Supplementary Figures S4 and S7).

When the stage of scCO_2_-assisted processing at the pressure of 15 MPa was introduced to the protocol, no significant improvement in the decellularization outcome was achieved (Supplementary Figure S3). The porosity of the valve leaflets increased, yet significantly less than after the alkaline treatment (Supplementary Figure S8). The elastic fiber packing in spongiosa and ventricularis was less dense than before scCO_2_-assisted processing (Supplementary Figure S6). Similar to the alkaline-based protocol, the valve leaflets acquired higher Young’s modulus and UTS as compared to the native tissue (Table 1). However, strain at fracture was comparable with the native sample. 

A pronounced decellularization devoid of accumulations of residual nuclear and membrane fragments of fibroblasts and smooth myocytes was achieved after the scCO_2_-assisted processing at the pressure of 25 MPa (Figure 1, Supplementary Figures S3 and S5). In contrast to the previous protocol employing the pressure of 15 MPa, at 25 MPa, the increased porosity appeared in the aortic walls, but not in the valve leaflets (Supplementary Figure S8). Young’s modulus of the valve leaflets also appeared lower (Table 1). 

Even though no significant differences in the distribution of collagen fibers in the aortic walls and valve leaflets were found at different pressure values, when higher pressure was applied, the elastic fibers of fibrosa became looser, while in spongiosa and ventricularis they remained intact. Elastic membranes were also looser in the media. The scCO_2_-assisted processing reduced the strain at fracture of the valve leaflets and Young’s modulus of the aortic walls at both pressure values (Table 1). 

The content of GAGs after the scCO_2_-assisted processing did not change (Supplementary Figures S4 and S7).

In order to confirm the removal of residual nuclear material following treatment with SDS/SD and processing in scCO_2_ medium (P = 25 MPa), we measured the DNA concentrations. We revealed that DNA content reduced from 260 ± 40 ng/mg in the native sample to 38 ± 4 ng/mg after the treatment with detergents alone and 26 ± 2 ng/mg with the following processing in scCO_2_ medium at the pressure of 25 MPa. 

**Figure 4:**
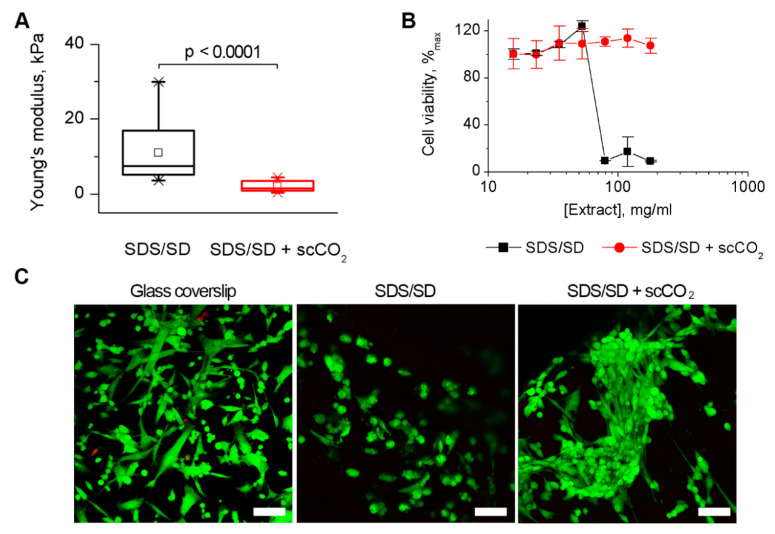
Mechanical characteristics and cytotoxicity of ovine aortic roots decellularized in a 0.5% sodium dodecyl sulfate/0.5% sodium deoxycholate detergent solution (SDS/SD) and after the subsequent processing in scCO_2_ medium (T = 37 °C, P = 25 MPa, t = 3 h). (**A**). Mechanical characteristics established by atomic force microscopy. Differences between the groups were analyzed by one-way Brown-Forsythe ANOVA followed by a post-hoc Tukey test. (**B**). [3-(4,5-dimethylthiazol-2-yl)-2,5-diphenyltetrazolium bromide] (MTT) test for the estimation of cytotoxicity towards 3T3 murine fibroblasts. (**C**). Confocal laser scanning microscopy of human umbilical cord multipotent mesenchymal stromal cells stained with calcein-AM (green) and ethidium homodimer-1 (red) after 48 h of culturing on the sample surfaces and a glass coverslip (control). Scale bar = 100 µm.

Since the mechanical properties of blood vessels cannot be fully described by a single uniaxial tensile test, we additionally performed indentation of the aortic walls after the detergent-based protocols with atomic force microscopy (AFM) that maps local mechanical characteristics via the compression of the sample surface. The measurement was performed in a liquid environment and revealed a decrease in Young’s modulus following the scCO_2_-assisted processing at 25 MPa (Figure 4A, Supplementary Figure S10). It is known that the mechanical properties of collagenous tissues are predetermined by the arrangement of the collagen fibrils [37]. The results of this study correlate with the increased porosity in the aortic walls revealed in histological study and uniaxial tensile test data (Supplementary Figure S8, Table 1). 

**Figure 5:**
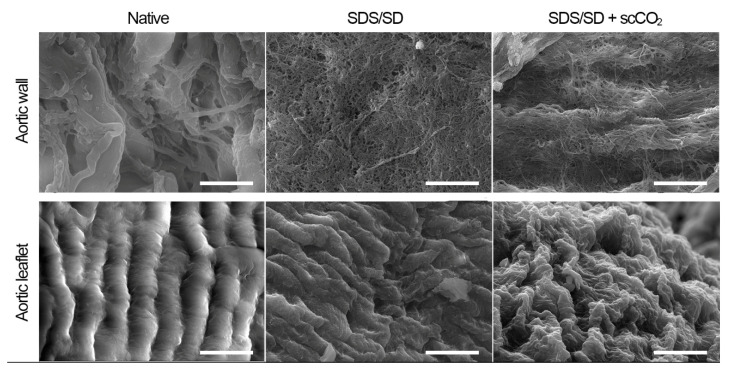
Scanning electron microscopy of ovine aortic roots decellularized in a 0.5% SDS/0.5% SD detergent solution (SDS/SD) and after the subsequent processing in scCO_2_ medium (T = 37 °C, P = 25 MPa, t = 3 h). Scale bar = 10 μm.

Regardless of the increased porosity, the preservation of the structural integrity of the ECM was confirmed by the scanning electron microscopy in both the aortic walls and valve leaflets following scCO_2_-assisted processing at 25 MPa (Figure 5).

Additionally, the cytotoxicity inherent to detergents was examined before and after the scCO_2_ treatment (P = 25 MPa). Figure 4B shows that scCO_2_ improved the cytocompatibility of the tissues by means of washing out residual detergents. Nonetheless, both samples were able to recellularize with human umbilical cord multipotent mesenchymal stromal cells (Figure 4C). The cells appeared morphologically healthier on the scCO_2_-processed sample.

## 3. Discussion

The fabrication of aortic valve bioprostheses requires a precise control of the structural-functional properties of the resulting product. Therefore, the decellularization procedure aiming at the elimination of the donor cell material must preserve the structural integrity of ECM with its inherent functions and cytocompatibility [38]. However, in complex 3D tissue structures, the decellularization is prohibited in dense or deep regions. The application of scCO_2_ is believed to improve the process performance. The approaches to decellularization based on the processing in scCO_2_ medium have been recently established for various tissues [39]. In our work, we aimed to evaluate the feasibility of existing protocols and their modifications for the aortic root decellularization. The object of our study was the ovine aortic root decellularized in various combinations of scCO_2_ medium with traditional decellularizing agents. The choice of the donor animal was due to the need for the most accurate modeling of immunological characteristics and hemodynamic parameters close to the human and high propensity for calcification desirable for pre-clinical trials [40]. Although ovine leaflet depth is shallower by about 2 mm than that of human, from a histomorphometrical point of view, they have similar annulus diameters and percent of leaflet contribution to circumference and do not have significant discrepancies in the commissure heights, spacing and leaflet free edges of individual leaflets [41]. 

Decellularized materials should not contain cellular components, including the cytoplasm and nuclei. Their presence in ECM can contribute to the violation of the biocompatibility and cause adverse reactions after the implantation. To assess the decellularization effectiveness and maintain the ECM structural integrity, we employed a histological analysis. Structural alterations of ECM under different decellularization conditions are of great importance because its components—collagen, elastin and GAGs—are responsible for the mechanical properties of bioprostheses, cell adhesion and proliferation [42]. In turn, biomechanical properties of heart valve prostheses predetermine their hemodynamic function [43]. Therefore, in our study, we also evaluated uniaxial tensile properties of the samples.

In [22], Sawada et al. in a model of porcine aorta, demonstrated the effectiveness of decellularization in scCO_2_ (15 = MPa, T = 37 °C, t = 20 min) with ethanol employed as a co-solvent. In our work, we adopted this protocol in the decellularization of a complex 3D structure, the ovine aortic root. The processing time was prolonged to 3 h since the effect of decellularization was not seen at a shorter time (data not shown). However, in both ethanol-based protocols, satisfactory decellularization was not achieved. Higher pressure (25 MPa) enabled only partial smooth muscle cell elimination; fibroblast removal also took place in the leaflets due to their thin-walled structures. In addition, this protocol promoted condensation of the collagen fibers in ventricularis. The density of elastic fibers in intima and spongiosa and elastic lamellae in media also increased and was greater than in most the protocols we used. This is in correspondence with the increased Young’s moduli of the aortic walls and leaflets. The value of UTS in the circumferential direction of aorta was also the highest among all the protocols. These results may be related to tissue dehydration due to the hygroscopic nature of ethanol. In [30], the dehydration of porcine aorta fragments was prevented by means of presaturating scCO_2_ with water before contacting the tissue. However, the addition of water to the ethanol did not markedly affect the extent of decellularization. In our study, a dramatic difference was found between the elastic fiber organization in fibrosa after scCO_2_-assisted processing at different pressure values. At 15 MPa, the fibers loosened, while, at 25 MPa, they condensed. Loosening of the fibers occurred in fibrosa due to the fragile structure of the leaflets. The increase in the pressure is believed to enhance dehydration by means of increased water uptake, so that the condensation appeared. The fiber condensation may promote the material contraction and reduce its porosity, thus compromising natural mechanical and hemodynamic properties and prohibiting cellular infiltration. Such alterations may cause late leaflet distortion and dysfunction. These findings match similar testing done in [36] that also showed the damage to the ovine aortic wall ECM at the higher pressure. These disadvantages limit the application of these protocols to the aortic root decellularization purposes. 

In contrast to the previous protocol, alkali-based decellularization provided substantial removal of all cell types from the aortic walls and valve leaflets depending on the pressure value. However, structural derangements (porosity) in both the aortic wall and valve leaflet ECM and deprivation of spongiosa were the hallmarks of this protocol, regardless of the pressure value (10 or 15 MPa). Denser collagen and elastin fiber packing in remaining fibrosa and ventricularis were noted after the treatment. Our results also show that the decellularization process lowers the GAGs content in the tissue. Even though in [28] this protocol enabled the decellularization of porcine and bovine pericardium without significant effect on its mechanical and structural properties, it appeared unsuitable for the decellularization of ovine aortic roots. The caustic nature of the alkali caused the loss of elasticity of the valve leaflet ECM that may promote overt fibrosis, detachment, or even apoptosis [44].

Finally, the detergent-based protocols were assessed using SDS/SD solution as a preconditioning medium followed by scCO_2_-assisted processing at the pressure of either 15 or 25 MPa. To begin with, we reproduced a state-of-the-art protocol of decellularization based on the detergent treatment. The extent of decellularization was comparable with that after the alkaline treatment. However, the inclusions of residual cellular material (fragments of cell membranes and nuclei) were identified in dense and deep structures, mostly between collagen fiber bundles in the pars fibrosa and spongiosa of the valve leaflets and aortic media. The decellularization process appeared to result in the most widespread distribution of elastic fibers in the spongiosa of the all the experimental groups. The collagen fiber packing was only affected in the valve leaflet fibrosa and spongiosa leading to the looser arrangement. The mechanical properties were close to that of native tissue and matched the results of the study [45]. These findings indicate relatively mild treatment conditions.

The processing in scCO_2_ medium at the pressure of 15 MPa improved the decellularization outcome, which is in accordance with the data obtained earlier for porcine aorta in [30]. However, the decellularization was still not complete; and the aortic valve leaflet porosity increased. No such disadvantages were found at 25 MPa. The value of Young’s modulus of the leaflets reached ~100 MPa and was much lower than that of the alkali-based treatment. However, relatively high Young’s modulus and UTS of the leaflets revealed in our study must be taken into consideration while manufacturing aortic valve bioprostheses. This is closely related to the decreased cross-section area of the leaflets as the result of decellularization. Following recellularization and the remodeling of the leaflets may affect the mechanical properties, bringing them closer to the native tissue [46]. After the scCO_2_-assisted processing, we achieved a satisfactory decellularization devoid of accumulations of residual nuclear and membrane fragments of fibroblasts and smooth myocytes. Smooth myocyte elimination appeared the most challenging due to their organization into bundles localized deeply in the aortic wall. The result was also supported with the measurement of residual DNA content after the detergent treatment alone (15% DNA remained) and following processing in scCO_2_ medium at 25 MPa (10% DNA remained). Elimination of at least 90% of the host DNA and the lack of visible nuclear material in tissue sections stained with a nuclear stain meets the minimal criteria that suffice to satisfy the intent of decellularization aiming to avoid serious immunological reaction and is crucial in post-implantation functionality [47]. 

Since the use of detergents is known to increase the cytotoxicity of tissue grafts [20], we carried out a comparative in vitro cytotoxicity study of the aortic valves decellularized in a detergent solution (SDS/SD) and after the subsequent processing in scCO_2_ medium (T = 37 °C, P = 25 MPa, t = 3 h). The dramatic decrease in cytotoxicity level was revealed after the processing, which is favorable for possible clinical application of this protocol. Additionally, human umbilical cord multipotent mesenchymal stromal cells were amenable for recellularization of the ECM.

A decrease in the Young’s modulus when measured by AFM correlated with the increase in the porosity in the aortic walls revealed in histological study. It is known that an increase in transplant porosity contributes to recellularization [48] and angiogenesis [49,50]. Yet, according to the scanning electron micrographs, the histoarchitecture of the valve leaflets was preserved.

To conclude, the present study was focused on establishing the effects of different scCO_2_-assisted protocols for the decellularization of ovine aortic roots on the cell elimination and ECM structure. The favorable effects were revealed for the protocol, including processing in scCO_2_ medium (T = 37 °C, P = 25 MPa, t = 3 h) and preconditioning in a detergent solution. The scCO_2_-assisted processing reduced cytotoxicity and improved the decellularization (endotheliocytes, fibroblasts and smooth myocytes) outcome of the ovine aortic roots. The extent of decellularization was greater compared to the ethanol-based treatments while the effects on the ECM structure and mechanical properties of aortic valve leaflets were lower than that of alkali-based treatments. The decellularization feasibility and disclosed effects of the methods described herein upon ECM structure/composition and mechanical properties of ovine aortic roots are intended as guidelines in the design and manufacture of effective aortic root allografts. Further studies will be necessary to determine the hemodynamic properties and bioprosthesis–host tissue integration. 

## 4. Materials and Methods

All chemicals were purchased from Sigma-Aldrich (Merck, Kenilworth, NJ, USA), unless otherwise specified. 

### 4.1. Isolation of the Aortic Root

The experiments were carried out after the approval of the local ethics committee (Sechenov University, Moscow, Russia). Aortic root decellularization was performed with 77 valve-containing fragments of the ovine aortas. Sheep organocomplexes were obtained at a local slaughterhouse and transported to the laboratory in a thermocontainer filled with sterile phosphate-buffered saline (PBS, EcoService, Saint Petersburg, Russia) at +4 °C. The isolation of the hearts from the organocomplexes was carried out under sterile conditions.

The procedure for isolating each aortic root was carried out in a laminar flow biological safety box, class II, Safe 2020 1.2 (Thermo Fisher Scientific, Waltham, MA, USA) in several stages. At the first stage, an access to the aortic root was provided. Then, the pericardium was opened and dissected, the main vessels were inspected. Surrounding tissues located distal to the great vessels, as well as around them, were removed. The aortopulmonary curtain was dissected, separating the aorta from the main pulmonary artery. Then, using scissors, connective tissue cords were sequentially separated, gradually approaching the aortic root. The pulmonary artery was removed. The coronary arteries were isolated. At the second stage, the excessive myocardium tissue was removed. To this purpose, the myocardium of the right ventricle and atrium was dissected. Next, the left atrial appendage was excised, the posterior leaflet was identified and the myocardium of the left ventricle was dissected along it. Then, the chords of the anterior mitral valve leaflet were removed and most of the myocardium was cut off, leaving only the basal part. Finally, with high precision steps, a muscle cuff of the aortic root was gradually formed. During the isolation process, the material was periodically irrigated with non-buffered isotonic saline (0.9% NaCl) to prevent tissue drying. Aortic valves without apparent macroscopic pathology were included in the study.

### 4.2. Aortic Root Decellularization

Decellularization was carried out within 4–6 h after the isolation of the aortic roots as summarized in Table 2. Additionally, 250 mL sealing glass containers (Simax, Czech Republic) were used for preconditioning in order to eliminate the risk of unwanted contamination. The aortic valves were rinsed with povidone-iodine solution (10% Betadine, Egis, Hungary) and dipped into the containers with a sterile preconditioning medium (see Table 2) at 25 °C under constant stirring at 200 rpm on an OS-20 orbital shaker (Biosan, Riga, Latvia) followed by washing cycles with PBS (12 h). The processing in scCO_2_ medium was performed using an Applied Separations unit (SCF Green Technology Spe-ed SFE, Applied Separations Inc., Allenton, PA, USA). The experimental setup is shown in Supplementary Figure S11. The processing was carried out under temperature-controlled conditions at 37 ± 0.2 °C, a pressure of 10–25 MPa and a flow rate of 3 ± 0.5 mL/min. A wet ovine aortic root was placed in a high-pressure reactor (working volume 100 mL), the CO_2_ chamber was filled up at a pressure of 6–7 MPa, then the thermostat was turned on and the temperature was set at 37 °C and the pressure was adjusted to 10–25 MPa (achievement of the operating parameters was about 20 min). Next, the fine adjustment valve set the flow rate to 3 mL/min. Under such conditions, the samples were kept for 1–3 h, then the thermostat was turned off and slowly, over 30 min, the pressure was reduced in order to avoid swelling and severe structural changes under abrupt depressurization.

### 4.3. Histological Analysis

Aortic walls and valve leaflets of 5 samples in each experimental group were harvested for the histological study. The tissue samples were fixed in a solution of 10% neutral formalin, dehydrated, embedded in paraffin and sectioned into 4 μm transverse sections (3 sections per block). The transverse sections were then stained with hematoxylin and eosin to evaluate the general histoarchitecture of the tissues. Toluidine blue staining was used to visualize GAGs, Picrosirius red was used for collagen fibers and Weigert’s resorcin fuchsin was used for elastic fibers and lamellae. After embedding into EZ-Mount™ mounting medium (Shandon, Pittsburgh, PA, USA), and covering with coverslips, the samples were studied with a Leica DM4000 B LED universal microscope, equipped with a Leica DFC7000 T camera running under LAS V4.8 software (Leica Microsystems, Heerbrugg, Switserland). The morphological analysis was performed using bright-field and polarized light microscopy techniques.

The morphometric study of the tissue structures was performed using bright-field microscopy images acquired in 10 representative and randomly selected fields of view (FOV, 400×). We analyzed the mean numbers of intact fibroblasts and endothelial and smooth muscle cells, the density of collagen fibers, elastic fibers and lamellae in a FOV in each layer of aortic wall and valve leaflets. The cell-type estimation was performed under the histological inspection of hematoxylin-eosin-stained tissue sections as described in [51] and illustrated in Supplementary Figure S1, since the immunostaining of membrane and cytoplasmic markers is unreliable for aggressively treated tissues. We counted the cells with nuclei and cytoplasm visible in a bright-field microscope. The collagen fiber preservation was assessed by the red staining with Picrosirius red under bright-field microscopy and by birefringent properties of collagen molecules under polarized light microscopy [52]. The density of collagen fibers and elastic structures was calculated as the ratio of their area to the area of each layer of aortic walls and valve leaflets by digital analysis with Adobe Photoshop CS6 software. The degree of porosity (histoarchitecture preservation) and metachromatism (GAGs content) of the scaffolds in the samples were also assessed by a semi-quantitative evaluation (Supplementary Table S1). The scoring was applied by three independent observers.

NucBlue^TM^ Fixed Cell Reagent (Invitrogen) was used for staining cell nuclei. The tissue sections were deparaffinized and stained for 30 min at 37 °C according to the manufacturer instructions followed by thorough rinsing with PBS. Fluoromount^TM^ Aqueous Mounting Medium (Sigma, St. Louis, MI, USA) was applied to the tissue sections then covered with a coverslip and sealed. 

### 4.4. Confocal Laser Scanning Microscopy

For confocal imaging, we used the LSM 880 Airyscan (Carl Zeiss, Jena, Germany) scanning laser confocal microscope equipped with an AiryScan module and GaAsP detector (Carl Zeiss, Jena, Germany). Z-stack images (maximum projection) were obtained with an EC Plan-Neofluar 10×/0.3 M27 objective (Carl Zeiss, Obercochen, Germany). Additionally, 405, 488 and 561 nm laser lines were used to detect NucBlue^TM^ Fixed Cell Reagent, calcein and ethidium homodimer-1, respectively. 

### 4.5. Uniaxial Tensile Tests

In order to test the strain at fracture, UTS and Young’s Modulus, a uniaxial tension was exerted on the fragments of the leaflets (in circumferential direction) and aortas (in circumferential and longitudinal directions) until their failure using a mechanical tester model Mach-1 v500c (Biomomentum, Laval, QC, Canada). The wet tissue fragments of the 8 groups (Table 2) were cut into a dog bone shape, as schematically illustrated in Supplementary Figure S12. Before each test, the mechanical tester was calibrated using patterns provided by the manufacturer. Both ends of the samples were fixed tightly in the clamps followed by gradual stretching at a controlled temperature (37 °C) by applying constant tension to the ends and recording the data on a personal computer. The withdrawal rate was 50 mm/min (with a pre-load of 0.05 N) until the breaking point was reached. The parameters were calculated from the stress-strain curves. 

### 4.6. DNA Quantification Test

To determine the DNA concentration, the samples of decellularized and native aortic tissues (*n* = 6) were freeze-dried and cut into fragments weighing ~5 mg. Fragments obtained from the same sites were digested in Collagenase A (from *Clostridium histolyticum*) solution (2.5 mg/mL) in a buffer (50 mM Tris-HCl, pH 7.4) containing 10 mM calcium chloride and 0.02 mg/mL sodium azide (OOO PanEco company, Moscow, Russia). The samples were then incubated at 37 °C with periodic stirring on an IKA MS 3 BASIC SHAKER vortex mixer (Sigma Aldrich, St. Louis, MI, USA). For the following isolation of DNA, a standard set of reagents (Evrogen, Moscow, Russia) was used in accordance with the recommendations of the manufacturer. The amount of DNA was measured using a QuantiFluor^TM^ dsDNA kit and Quantus^TM^ fluorimeter (Promega, Madison, WI, USA).

### 4.7. MTT Test

MTT test was adopted from ISO 10993-5 in order to evaluate the effect of soluble components of decellularized ovine aortic roots on the viability of 3T3 murine fibroblasts. Tissue specimens were freeze-dried, sterilized by γ-radiation and incubated in 1 mL (180 mg dry weight) of DMEM/F12 culture medium supplemented with 100 U/mL streptomycin, 100 g/mL penicillin, 1% (*v*/*v*) GlutaMAX (Gibco), 5% (*v*/*v*) fetal bovine serum (HyClone) for 24 h at 37 °C. Serial dilutions of the obtained extracts in the culture medium were added in triplicates (100 μL) to a subconfluent monolayer of 3T3 murine fibroblasts in 96-well plates. Culture medium alone was used as a control. The plates were further incubated for 24 h at 37 °C in 5% (*v*/*v*) CO_2_ in air. For the MTT test, the extract and control media were replaced with 100 μL of the MTT solution (0.5 mg/mL in the culture medium without supplements) followed by the incubation in a CO_2_-incubator at 37 °C for 3 h. After discarding the MTT solution, 100 μL aliquots of dimethyl sulfoxide were added to all the wells and shaken. The color developed was quantified by measuring absorbance at 567 nm (reference, 650 nm) using a microplate photometer (*Multiscan FC*, Thermo Fisher Scientific, Waltham, MA, USA). 

### 4.8. Contact Cytotoxicity

Human multipotent mesenchymal stromal cells were harvested from Wharton’s jelly of an umbilical cord collected at the clinical facilities of Sechenov University after the woman signed an informed consent form. The study was conducted in accordance with the Declaration of Helsinki and all procedures were approved by the local ethics committee of Sechenov University (#07–17 from 13.09.2017, Moscow, Russia). The primary culture of the cells was obtained via the explant method. The umbilical cord samples were rinsed with Hank’s solution with antibiotics (100 U/mL penicillin and 100 μg/mL streptomycin) then cut longitudinally. The blood vessels were removed, and Wharton’s jelly was isolated and mechanically minced with scissors. The small Wharton’s jelly fragments (<1 mm) were placed into Petri dishes and covered with DMEM/F-12 medium (1:1; OOO PanEco company, Moscow, Russia) supplemented with L-glutamine (2 mmol/L; OOO PanEco company, Moscow, Russia), bFGF (20 ng/mL; Prospec, Rehovot, Israel), gentamicin (50 μg/mL; OOO PanEco company, Moscow, Russia), 1% insulin-selenite-transferrin (100×, BioloT, Moscow, Russia), and 10% FCS (HyClone, St. Louis, MI, USA). Cell morphology and immunophenotype were routinely checked with a phase-contrast microscope Primovert (Carl Zeiss, Oberkochen, Germany) and microfluidic cell sorter Sony SH800 (Sony Biotechnology, San Jose, CA, USA), respectively. The following anti-human antibodies were used for the flow cytometry immunophenotyping: CD44, CD105, CD90, CD73, CD45, CD34, CD11b, CD19 and HLA-DR, all conjugated with phycoerythrin (PE) and obtained from Miltenyi Biotec, Germany. Each sample included 20.000 events, and 3 different cell population samples from passage 4 were tested. Typical flow cytometry immunophenotyping results are presented in Supplementary Figure S13. After passage 4, the cells were seeded on the γ- sterilized aortic wall fragments in a 24-well plate (5 × 10^4^ cells per well). The cultivation was performed in 5% (*v*/*v*) CO_2_ with saturated humidity at 37 °C. After 48 h in culture, the cells were stained with a dye set, calcein-AM/ethidium homodimer-1 Live/Dead Viability/Cytotoxicity Kit for mammalian cells, then rinsed with PBS thrice and observed as described in Section 4.4 in order to visualize the green live cells and the red dead cells. 

### 4.9. Scanning Electron Microscopy

A standard sample preparation protocol was used as follows. After washing a cut block in PBS, it was incubated in buffered 2.5% glutaraldehyde for 24 h. After the fixation, the samples were washed again in PBS and dehydrated through an ascending ethanol gradient (30%, 40%, 50%, 60%, 70% and “absolute”). Final dehydration was achieved by exposure to acetone and critical-point drying. After the dehydration, a metallic conductive film (Au-Pd, 50–100 nm) was applied onto the surface of the samples by the plasma spraying. The surface structure of the samples was visualized using a Zeiss EVO LS10 scanning electron microscope (Zeiss, Oberkochen, Germany) operating under high vacuum. 

### 4.10. Atomic Force Microscopy

The local mechanical characteristics of the aortic walls were evaluated with an atomic force microscope (BioScope Resolve, Bruker, Billerica, MA, USA) in the Force Volume mode, in PBS at room temperature. All the images were acquired using the same cantilever with a spherical tip (sQUBE, Bickenbach, Germany), a 5-µm-diameter borosilicate glass sphere attached to the triangular probe (with the spring constant k = 0.0551 N/m, length of 200 µm, width of 2 × 28 µm, Cr/Au coating). A force curve acquisition rate of 5 Hz, a 32 × 32 points resolution of maps, and a scan size of 5 × 5 µm were used. The mean value of the Young’s modulus was calculated based on 26–29 measurements for each sample group (*n* = 5) using the Hertz model. The Nanoscope Analysis software (Bruker, Billerica, MA, USA) was used for the curve processing. 

### 4.11. Statistical Analysis

Statistical data analysis was performed using JASP software v0.11.1 (University of Amsterdam, Amsterdam, The Netherlands), KNIME v4.1.0, GraphPad Prism v8.4. Descriptive statistics minimum, maximum, mean ± SD, median (from 25 to 75 percentile) were calculated and Shapiro-Wilk test was performed. Spearman correlation coefficient was calculated for groups. For normally distributed data, the one-way Brown—Forsythe ANOVA followed by post-hoc Dunnett’s T3 test was performed; in other cases the Kruskal—Wallis test was used, followed by post-hoc Dunn’s test. Graphs are presented as box plots with whiskers (Tukey). Significance of differences was considered at *p* value < 0.05.

## 5. Conclusions

In the course of our experiments, we evaluated the effects of different protocols for the decellularization of ovine aortic roots based on various combinations of treatment in alkaline, ethanol or detergent solutions with scCO_2_-assisted processing. The ethanol-based protocols did not suffice in decellularization, while alkaline treatment deranged the ECM organization that can lead to the valve dysfunction, as a result of hemodynamic shifts and thromboembolism after implantation. These features make these protocols unsafe. However, favorable effects were demonstrated for the ovine aortic root decellularization by means of preconditioning in a detergent solution followed by scCO_2_-assisted processing at the pressure of 25 MPa. They included satisfactory decellularization; low cytotoxicity; preservation of the histoarchitecture of ECM of the aortic valve leaflets and an increase in the porosity of the aortic walls. However, UTS and Young’s modulus of aortic valve leaflets increased following the processing in scCO_2_ medium in all experimental groups that mast be taken into consideration while manufacturing aortic valve bioprostheses.

## Figures and Tables

**Table 1:** Thickness and mechanical properties of native and decellularized ovine aortic valve leaflets and walls measured from stress-strain curves obtained via uniaxial tensile testing.

Treatment Method	Tissue	Thickness, mm	Direction	Ultimate Tensile Strength, MPa	Strain at Fracture, %	Young’s Modulus, MPa
**None**	Leaflet	0.23 ± 0.04	Circ.*	4.19 ± 1.44	56.3 ± 14.4	12.01 ± 1.08
	Wall	1.5 ± 0.35	Circ.	0.43 ± 0.20	29.4 ± 5.0	2.83 ± 1.16
			Long.**	0.35 ± 0.11	36.6 ± 15.1	2.71 ± 1.37
**scCO_2_ (15 MPa)-Ethanol**	Leaflet	0.13 ± 0.01	Circ.	5.99 ± 1.54	22.5 ± 11.7	109.13 ± 15.24
	Wall	1.15 ± 0.17	Circ.	1.13 ± 0.20	55.0 ± 20.0	4.02 ± 2.02
			Long.	0.23 ± 0.04	18.5 ± 6.2	3.63 ± 0.93
**scCO_2_ (25 MPa)-Ethanol**	Leaflet	0.19 ± 0.04	Circ.	12.76 ± 6.09	31.5 ± 15.0	104.43 ± 29.57
	Wall	1.66 ± 0.21	Circ.	1.01 ± 0.58	54.2 ± 14.4	3.85 ± 0.68
			Long.	0.31 ± 0.10	26.7 ± 12.2	3.99 ± 0.76
**Alkali-salt + scCO_2_ (10 MPa)**	Leaflet	0.10 ± 0.01	Circ.	24.50 ± 9.19	46.6 ± 25.5	2043.60 ± 903.98
	Wall	1.30 ± 0.25	Circ.	0.46 ± 0.08	55.0 ± 25.0	1.04 ± 0.46
			Long.	0.14 ± 0.04	32.5 ± 7.5	0.90 ± 0.18
**Alkali-salt + scCO_2_ (15 MPa)**	Leaflet	0.10 ± 0.01	Circ.	47.38 ± 18.36	53.3 ± 24.4	4500.00 ± 2039.12
	Wall	1.42 ± 0.33	Circ.	0.60 ± 0.13	36.3 ± 3.8	1.62 ± 0.49
			Long.	0.19 ± 0.01	37.5 ± 5.0	0.69 ± 0.04
**Detergent**	Leaflet	0.15 ± 0.03	Circ.	5.52 ± 2.28	68.4 ± 32.3	4.79 ± 2.25
	Wall	1.26 ± 0.11	Circ.	0.48 ± 0.19	76.0 ± 21.7	0.31 ± 0.04
			Long.	1.27 ± 0.08	41.7 ± 17.2	2.46 ± 0.59
**Detergents + scCO_2_ (15 MPa)**	Leaflet	0.12 ± 0.03	Circ.	76.27 ± 27.24	15.0 ± 5.0	207.98 ± 38.54
	Wall	1.41 ± 0.15	Circ.	0.39 ± 0.06	22.5 ± 8.3	1.40 ± 0.67
			Long.	0.15 ± 0.04	42.5 ± 10.0	0.44 ± 0.06
**Detergents + scCO_2_ (25 MPa)**	Leaflet	0.12 ± 0.01	Circ.	20.43 ± 8.54	25.0 ± 5.0	106.20 ± 56.11
	Wall	1.16 ± 0.25	Circ.	0.87 ± 0.03	40.0 ± 10.0	1.15 ± 0.55
			Long.	0.15 ± 0.04	56.7 ± 32.2	0.51 ± 0.27

* Circumferential; ** Longitudinal.

**Table 2:** Protocols for the decellularization of ovine aortic roots.

Treatment Method	Ref.	Preconditioning	Extraction Parameters	Co-Solvent
scCO_2_-Ethanol	[22]	None	T = 37 °C, P = 15 MPa, t = 3 h	95% Ethanol
			T = 37 °C, P = 25 MPa, t = 3 h	95% Ethanol
Alkali-salt + scCO_2_	[28]	1 M NaOH + 0.8 M Na_2_SO_4_ (1 h)	T = 37 °C, P = 10 MPa, t = 1 h	None
			T = 37 °C, P = 15 MPa, t = 1 h	None
Detergent	[8]	0.5% SDS/0.5% SD (12 h)	-	None
Detergent + scCO_2_	[28,30]	0.5% SDS/0.5% SD (12 h)	T = 37 °C, P = 15 MPa, t = 3 h	None
			T = 37 °C, P = 25 MPa, t = 3 h	None

The control groups included untreated native samples (negative control) and decellularized in detergent solution according to a state-of-the-art protocol [8] (positive control).

## Supplementary Materials

The following are available online, Table S1. A histological semiquantitative scoring system for the evaluation of the degree of porosity and metachromatism of tissue samples; Figure S1. Histological study of native (**A1–3**) and decellularized ovine aortic roots under hematoxylin-eosin staining. The cellular inclusions are pointed out with arrowheads for endothelial cells, arrows for fibroblasts and asterisks (*) for smooth muscle cells. Decellularization protocols included scCO_2_-assisted processing at the pressure of 15 MPa (**B1–3**) and 25 MPa (**C1–3**) with the use of ethanol as a co-solvent; combinations of alkaline treatment with scCO_2_-assisted processing at the pressure of 10 MPa (**D1–3**) and 15 MPa (**E1–3**); detergents alone (**F1–3**) or with following scCO_2_ treatment at the pressure of 15 MPa (**G1–3**) and 25 MPa (**H1–3**). In all decellularization protocols, the general structure of each cell type in such accumulations was preserved, but there were uneven signs of destruction of nuclei (karyopyknosis, karyorhexis and karyolysis) and cytoplasm in some cells. Scale bar = 40 µm; Figure S2. The optical properties of collagen in aortic valve leaflets and walls of native (**A1–4**) and decellularized ovine aortic roots. Decellularization protocols included scCO_2_-assisted processing at the pressure of 15 MPa (**B1–4**) and 25 MPa (**C1–4**) with the use of ethanol as a co-solvent; combinations of alkaline treatment with scCO_2_-assisted processing at the pressure of 10 MPa (**D1–4**) and 15 MPa (**E1–4**); detergents alone (**F1–4**) or with following scCO_2_ treatment at the pressure of 15 MPa (**G1–4**) and 25 MPa (**H1–4**). The collagen fibers appeared bright in fibrosa (F), ventricularis (V), spongiosa (S) of valve leaflets and in intima (I), media (M) and adventitia (A) of aortas. Picrosirius red staining, polarized-light microscopy. Dashed lines mark the areas where 400× images were acquired. 100× scale bar = 100 µm; 400× scale bar = 300 µm; Figure S3. NucBlue^TM^ Fixed Cell Reagent-assisted confocal laser scanning microscopy of residual nuclei in aortic valve leaflets (**A1-H1**) and walls (**A2-H2**) of native (**A1–2**) and decellularized ovine aortic roots. Decellularization protocols included scCO_2_-assisted processing at the pressure of 15 MPa (**B1–2**) and 25 MPa (**C1–2**) with the use of ethanol as a co-solvent; combinations of alkaline treatment with scCO_2_-assisted processing at the pressure of 10 MPa (**D1–2**) and 15 MPa (**E1–2**); detergents alone (**F1–2**) or with following scCO_2_ treatment at the pressure of 15 MPa (**G1–2**) and 25 MPa (**H1–2**). Scale bar = 100 µm; Figure S4. The content of glycosaminoglycans (GAGs) in aortic valve leaflets and walls of native (**A1–4**) and decellularized ovine aortic roots. Decellularization protocols included scCO_2_-assisted processing at the pressure of 15 MPa (**B1–4**) and 25 MPa (**C1–4**) with the use of ethanol as a co-solvent; combinations of alkaline treatment with scCO_2_-assisted processing at the pressure of 10 MPa (**D1–4**) and 15 MPa (**E1–4**); detergents alone (**F1–4**) or with following scCO_2_ treatment at the pressure of 15 MPa (**G1–4**) and 25 MPa (**H1–4**). The GAGs had pink or purple color (metachromatism); there were prominent GAGs content in fibrosa (F) and media (M) in control group; in the same layers in other groups and in ventricularis (V), spongiosa (S), intima (I) and adventitia (A) in all groups the coloration was scarce. Toluidine blue staining. Dashed lines mark the areas where 400× images were acquired. 100× scale bar = 100 µm; 400× scale bar = 300 µm; Figure S5. The content of endotheliocytes, fibroblasts and smooth myocytes in aortic valve leaflets and walls of native (**A**) and decellularized ovine aortic roots. Decellularization protocols included scCO_2_-assisted processing at the pressure of 15 MPa (**B**) and 25 MPa (**C**) with the use of ethanol as a co-solvent; combinations of alkaline treatment with scCO_2_-assisted processing at the pressure of 10 MPa (**D**) and 15 MPa (**E**); detergents alone (**F**) or with following scCO_2_ treatment at the pressure of 15 MPa (**G**) and 25 MPa (**H**). Differences between the groups were analyzed by Kruskal–Wallis test followed by post-hoc Dunn’s test. Middle horizontal line in each box with whiskers (Tukey) represents the median, dots represent extreme points. * *p* < 0.05, ** *p* < 0.005, *** *p* < 0.001, **** *p* < 0.0001 vs. native sample (**A**); Figure S6. The density of collagen and elastin in aortic valve leaflets and walls of native (**A**) and decellularized ovine aortic roots. Decellularization protocols included scCO_2_-assisted processing at the pressure of 15 MPa (**B**) and 25 MPa (**C**) with the use of ethanol as a co-solvent; combinations of alkaline treatment with scCO_2_-assisted processing at the pressure of 10 MPa (**D**) and 15 MPa (**E**); detergents alone (**F**) or with following scCO_2_ treatment at the pressure of 15 MPa (**G**) and 25 MPa (**H**). Differences between the groups were analyzed by one-way Brown—Forsythe ANOVA followed by Dunnett’s T3 post-hoc test (for collagen in fibrosa, spongiosa, intima, adventitia and elastin in ventricularis, intima) or Kruskal—Wallis test followed by post-hoc Dunn’s test (for collagen in ventricularis, media and elastin in fibrosa, spongiosa, media, adventitia). Middle horizontal line in each box with whiskers (Tukey) represents the median, dots represent extreme points. * *p* < 0.05, ** *p* < 0.005, *** *p* < 0.001, **** *p* < 0.0001 vs. native sample (**A**); Figure S7. Toluidine blue-assisted metachromatism of aortic valve leaflets and walls of native (**A**) and decellularized ovine aortic roots scored with score 0—none, score 1—weak, score 2—pronounced. Decellularization protocols included scCO_2_-assisted processing at the pressure of 15 MPa (**B**) and 25 MPa (**C**) with the use of ethanol as a co-solvent; combinations of alkaline treatment with scCO_2_-assisted processing at the pressure of 10 MPa (**D**) and 15 MPa (**E**); detergents alone (**F**) or with following scCO_2_ treatment at the pressure of 15 MPa (**G**) and 25 MPa (**H**). Differences between the groups were analyzed by Kruskal—Wallis test followed by post-hoc Dunn’s test. Middle horizontal line in each box with whiskers (Tukey) represents the median, dots represent extreme points. * *p* < 0.05, ** *p* < 0.005, *** *p* < 0.001, **** *p* < 0.0001 vs. native sample (**A**); Figure S8. The porosity of aortic valve leaflets and walls of native (**A**) and decellularized ovine aortic roots scored with score 0—none, score 1—weak, score 2—pronounced. Decellularization protocols included scCO_2_-assisted processing at the pressure of 15 MPa (**B**) and 25 MPa (**C**) with the use of ethanol as a co-solvent; combinations of alkaline treatment with scCO_2_-assisted processing at the pressure of 10 MPa (**D**) and 15 MPa (**E**); detergents alone (**F**) or with following scCO_2_ treatment at the pressure of 15 MPa (**G**) and 25 MPa (**H**). Differences between the groups were analyzed by Kruskal—Wallis test followed by post-hoc Dunn’s test. Middle horizontal line in each box with whiskers (Tukey) represents the median, dots represent extreme points. * *p* < 0.05, ** *p* < 0.005, *** *p* < 0.001, **** *p* < 0.0001 vs. native sample (A); Figure S9. The gross appearance of native (**A**) and decellularized ovine aortic roots. Decellularization protocols included scCO_2_-assisted processing at the pressure of 15 MPa (**B**) and 25 MPa (**C**) with the use of ethanol as a co-solvent; combinations of alkaline treatment with scCO_2_-assisted processing at the pressure of 10 MPa (**D**) and 15 MPa (**E**); detergents alone (**F**) or with following scCO_2_ treatment at the pressure of 15 MPa (**G**) and 25 MPa (**H**); Figure S10. Mapping the local Young’s modulus of ovine aortic roots decellularized in a 0.5% SDS/0.5% SD detergent solution (**A1–4**) and after the subsequent processing in scCO_2_ medium (T = 37 °C, P = 25 MPa, t = 3 h) (**B1–4**) using atomic force microscopy; Figure S11. The experimental setup (1—cylinder with CO_2_, 2—pump, 3—reactor thermostat, 4—regulating valve thermostat, 5—reactor, 6—fine adjustment valve); Figure S12. Schematic representation of the sample preparation for uniaxial tensile testing; Figure S13. Characterization of the human umbilical cord multipotent mesenchymal stromal cells by flow cytometry. The cells (passage 4) were stained for the specific surface markers as described in Materials and Methods section. Expanded cells demonstrated the expression of CD44, CD105, CD90, CD73 and were negative for CD45, CD34, CD11b, CD19 and HLA-DR (green). Isotype controls conjugated with PE (blue) was run for the comparison.
