# Supplementary material for: Evaluation of Supercritical CO2-Assisted Protocols in a Model of Ovine Aortic Root Decellularization
Source: Molecules. 2020 Aug 27;25(17):3923. doi: 10.3390/molecules25173923 (PMC7504408; doi:10.3390/molecules25173923)
Supplement: Supplementary file 1 [file molecules-25-03923-s001.pdf]

Evaluation of supercritical CO_2-assisted protocols in a model of ovine aortic root decellularization

Elvira R. Gafarova [1,*], Ekaterina A. Grebenik [1], Alexey E. Lazhko [2], Anastasia A. Frolova [1], Anastasia S. Kuryanova [1,3], Alexandr V. Kurkov [1], Ilya A. Bazhanov [1], Byron S. Kapomba [1], Nastasia V. Kosheleva [4-5], Ivan A. Novikov [6], Anatoly B. Shekhter [1], Elena N. Golubeva [7], Anna B. Soloviova [3] and Peter S. Timashev [1,3,7]

[1] Institute for Regenerative Medicine, Sechenov First Moscow State Medical University, 119991, Moscow, Russia;
[2] Kurnakov Institute of General and Inorganic Chemistry, Russian Academy of Sciences, 119991, Moscow, Russia;
[3] N. N. Semenov Federal Research Center for Chemical Physics, Russian Academy of Sciences, 117977, Moscow, Russia;
[4] FSBSI "Institute of General Pathology and Pathophysiology", 125315, Moscow, Russia;
[5] Faculty of Biology, Lomonosov Moscow State University, 119234, Moscow, Russia
[6] Scientific Research Institute of Eye Diseases, 119021 Moscow, Russia;
[7] Faculty of Chemistry, Lomonosov Moscow State University, 119991, Moscow, Russia;

* Correspondence: gafarova.elv@gmail.com, Tel.: +7-917-372-5217

Table S1. A histological semiquantitative scoring system for the evaluation of the degree of porosity and metachromatism of tissue samples.

Points	**Changes in a sample (sample porosity* / sample metachromatism**) in each FOV (400×)**	**Examples**
0	No change or weak focal changes in less than 25% of the sample area	Figure 1 (A1-A2), Supplementary Figure S4 (C1-C2)
1	Weak focal changes in more than 25-50% of the sample area	Figure 1 (G1-G2), Supplementary Figure S4 (C3-C4)
2	Pronounced focal or weak/pronounced diffuse changes in more than 25% of the sample area	Figure 1 (D1-D2, E1-E2), Supplementary Figure S4 (A1-A4)

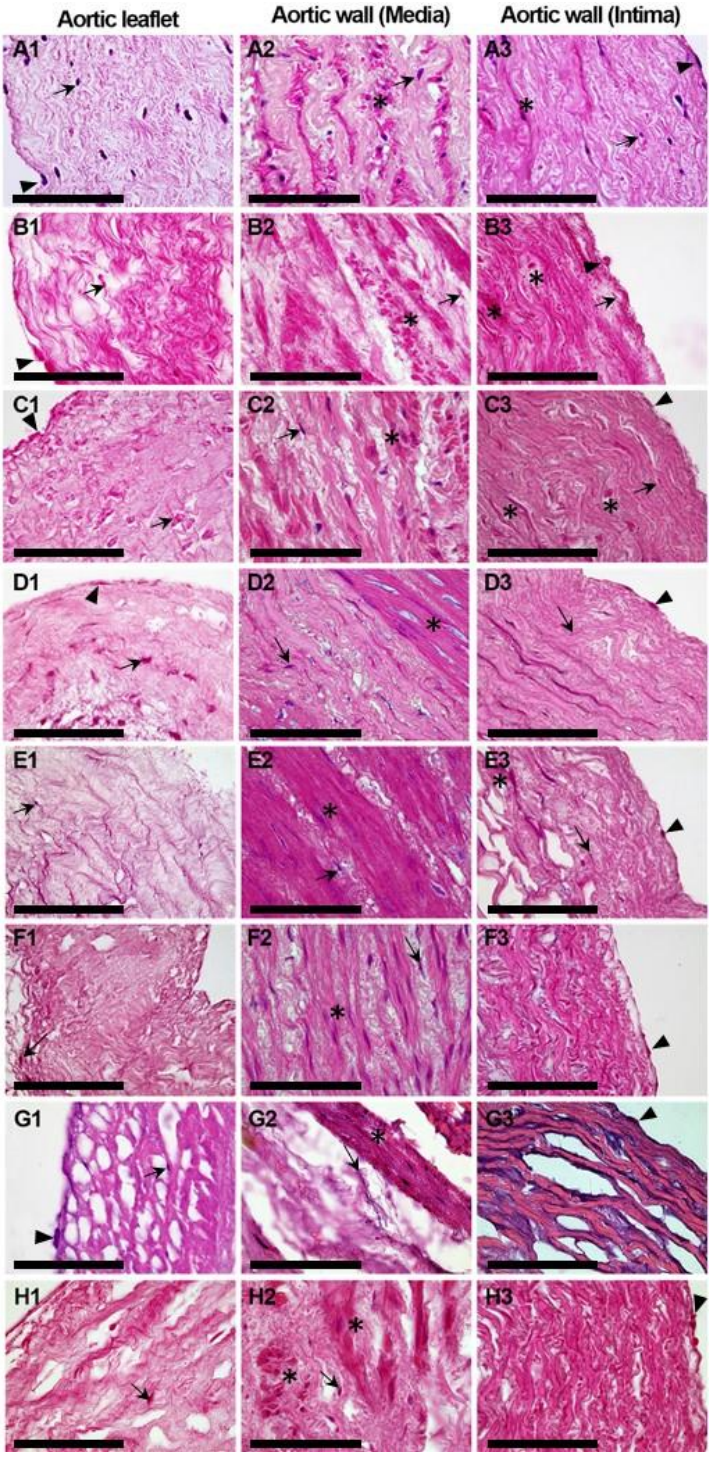

Figure S1. Histological study of native **(A1-3)** and decellularized ovine aortic roots under hematoxylin-eosin staining. The cellular inclusions are pointed out with arrowheads for endothelial cells, arrows for fibroblasts and asterisks (*) for smooth muscle cells. Decellularization protocols included $scCO_2$-assisted processing at the pressure of 15 MPa **(B1-3)** and 25 MPa **(C1-3)** with the use of ethanol as a co-solvent; combinations of alkaline treatment with $scCO_2$-assisted processing at the pressure of 10 MPa **(D1-3)** and 15 MPa **(E1-3)**; detergents alone **(F1-3)** or with following $scCO_2$ treatment at the pressure of 15 MPa **(G1-3)** and 25 MPa **(H1-3)**. In all decellularization protocols, the general structure of each cell type in such accumulations was preserved, but there were uneven signs of destruction of nuclei (karyopyknosis, karyorhexis and karyolysis) and cytoplasm in some cells. Scale bar = 40 μm.

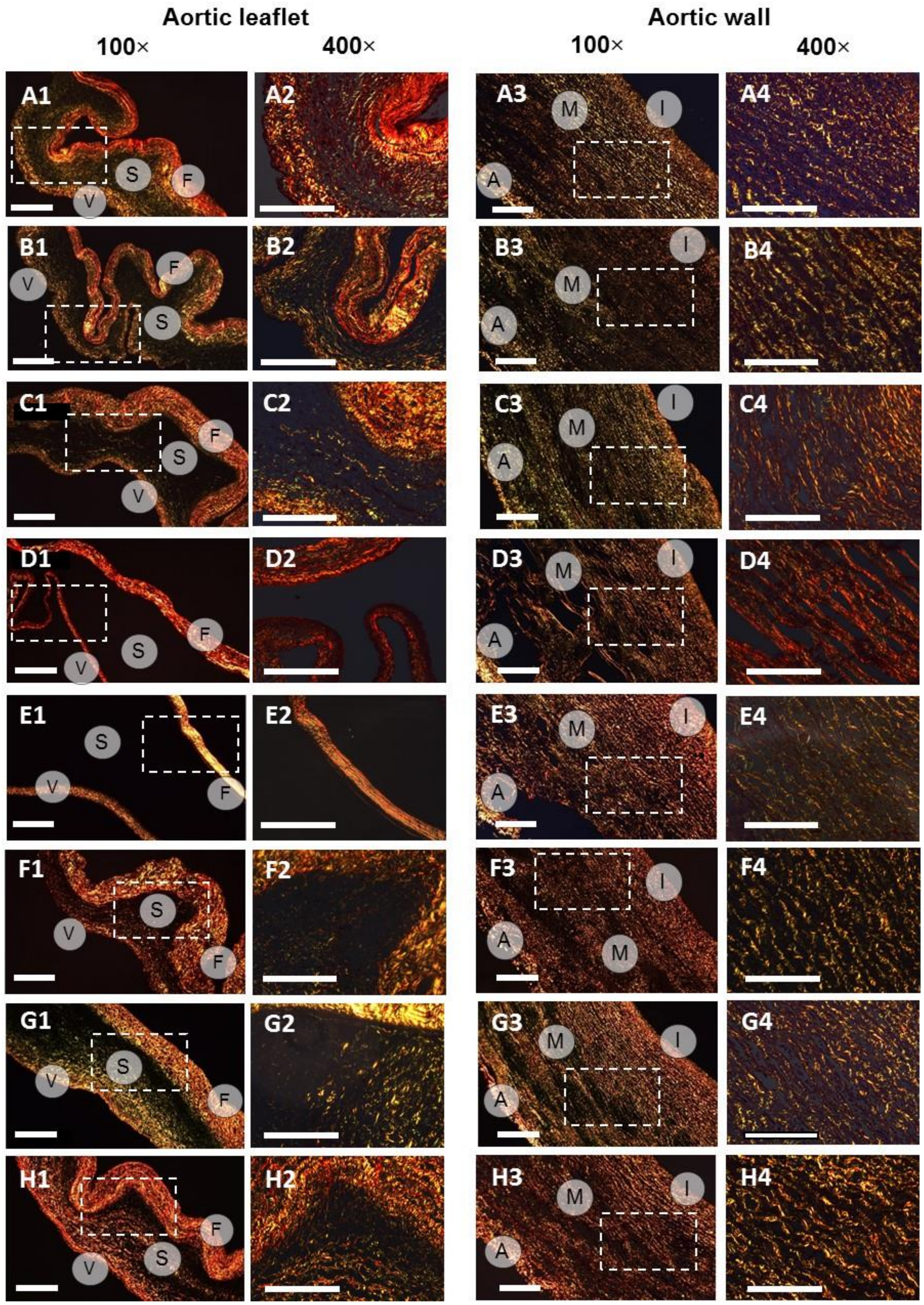

Figure S2. The optical properties of collagen in aortic valve leaflets and walls of native (**A1-4**) and decellularized ovine aortic roots. Decellularization protocols included $scCO_2$-assisted processing at the pressure of 15 MPa **(B1-4)** and 25 MPa **(C1-4)** with the use of ethanol as a co-solvent; combinations of alkaline treatment with $scCO_2$-assisted processing at the pressure of 10 MPa **(D1-4)** and 15 MPa **(E1-4)**; detergents alone **(F1-4)** or with following $scCO_2$ treatment at the pressure of 15 MPa **(G1-4)** and 25 MPa **(H1-4)**. The

collagen fibers appeared bright in fibrosa (F), ventricularis (V), spongiosa (S) of valve leaflets and in intima (I), media (M) and adventitia (A) of aortas. Picrosirius red staining, polarized-light microscopy. Dashed lines mark the areas where 400× images were acquired. 100× scale bar = 100 μm; 400× scale bar = 300 μm.

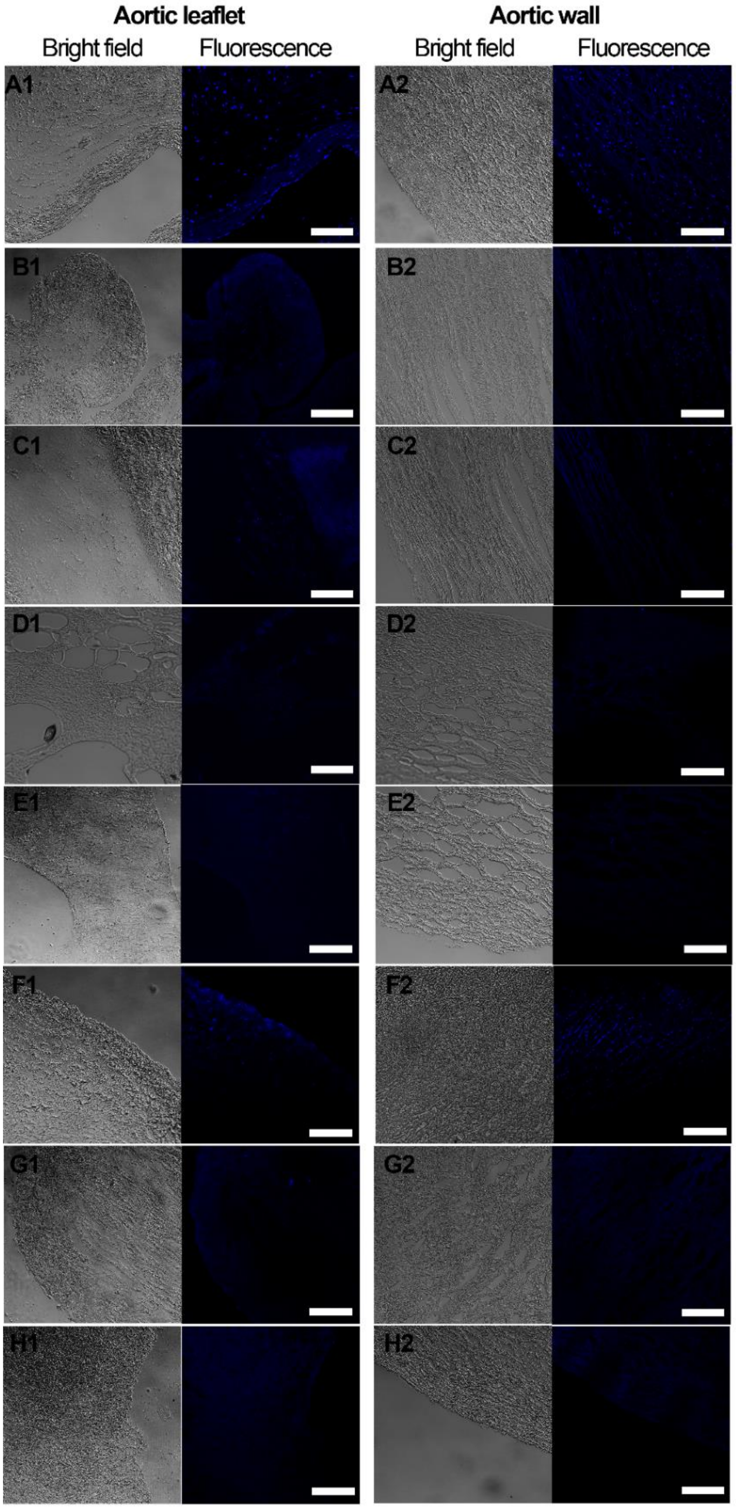

Figure S3. NucBlue™ Fixed Cell Reagent-assisted confocal laser scanning microscopy of residual nuclei in aortic valve leaflets **(A1-H1)** and walls **(A2-H2)** of native **(A1-2)** and decellularized ovine aortic roots. Decellularization protocols included $scCO_2$-assisted processing at the pressure of 15 MPa **(B1-2)** and 25 MPa **(C1-2)** with the use of ethanol as a co-solvent; combinations of alkaline treatment with $scCO_2$-assisted

processing at the pressure of 10 MPa **(D1-2)** and 15 MPa **(E1-2)**; detergents alone **(F1-2)** or with following $scCO_2$ treatment at the pressure of 15 MPa **(G1-2)** and 25 MPa **(H1-2)**. Scale bar = 100 μm.

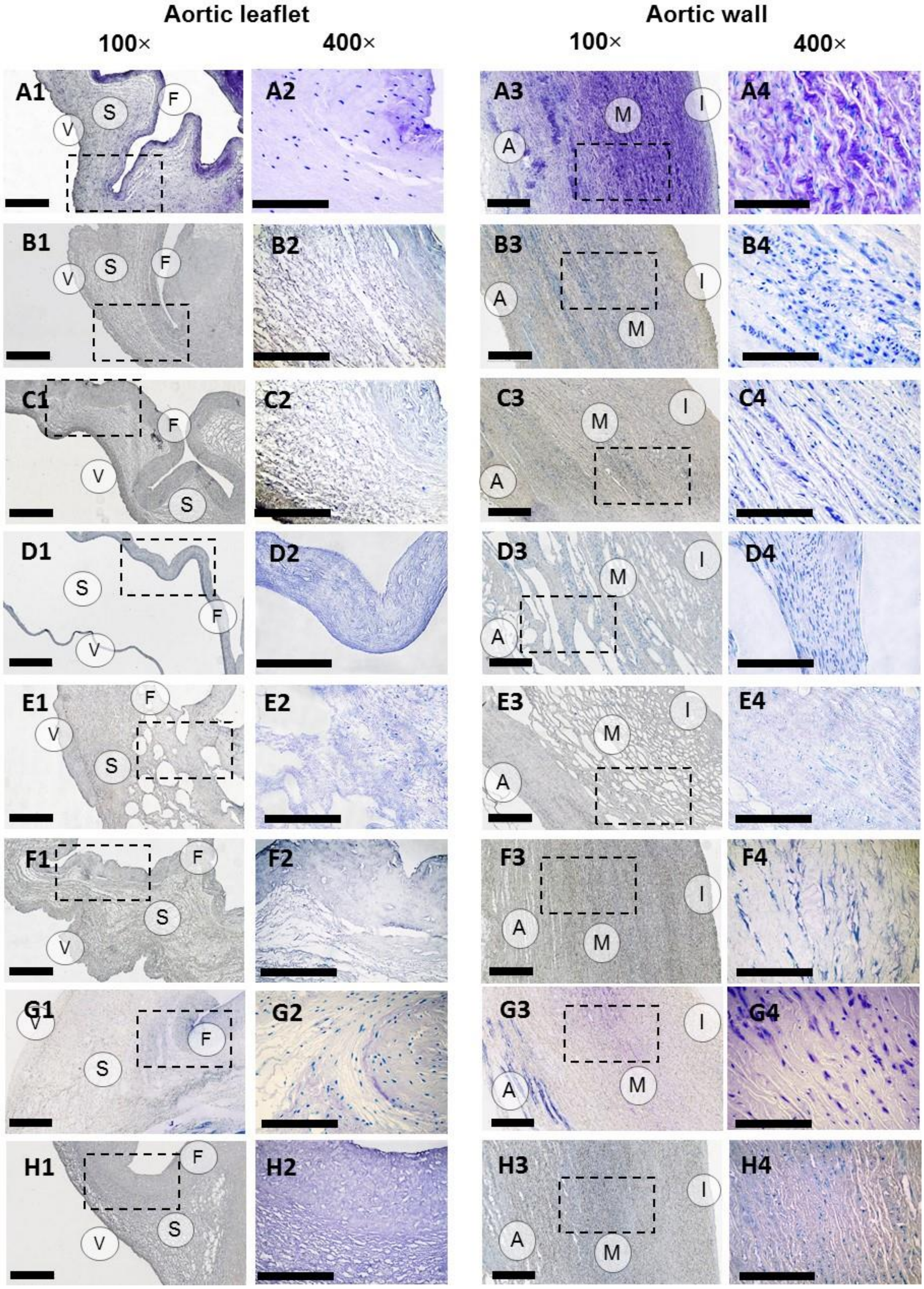

Figure S4. The content of glycosaminoglycans (GAGs) in aortic valve leaflets and walls of native (**A1-4**) and decellularized ovine aortic roots. Decellularization protocols included $scCO_2$-assisted processing at the pressure of 15 MPa **(B1-4)** and 25 MPa **(C1-4)** with the use of ethanol as a co-solvent; combinations of alkaline

treatment with scCO$_2$-assisted processing at the pressure of 10 MPa **(D1-4)** and 15 MPa **(E1-4)**; detergents alone **(F1-4)** or with following scCO$_2$ treatment at the pressure of 15 MPa **(G1-4)** and 25 MPa **(H1-4)**. The GAGs had pink or purple color (metachromatism); there were prominent GAGs content in fibrosa (F) and media (M) in control group; in the same layers in other groups and in ventricularis (V), spongiosa (S), intima (I) and adventitia (A) in all groups the coloration was scarce. Toluidine blue staining. Dashed lines mark the areas where 400× images were acquired. 100× scale bar = 100 μm; 400× scale bar = 300 μm.

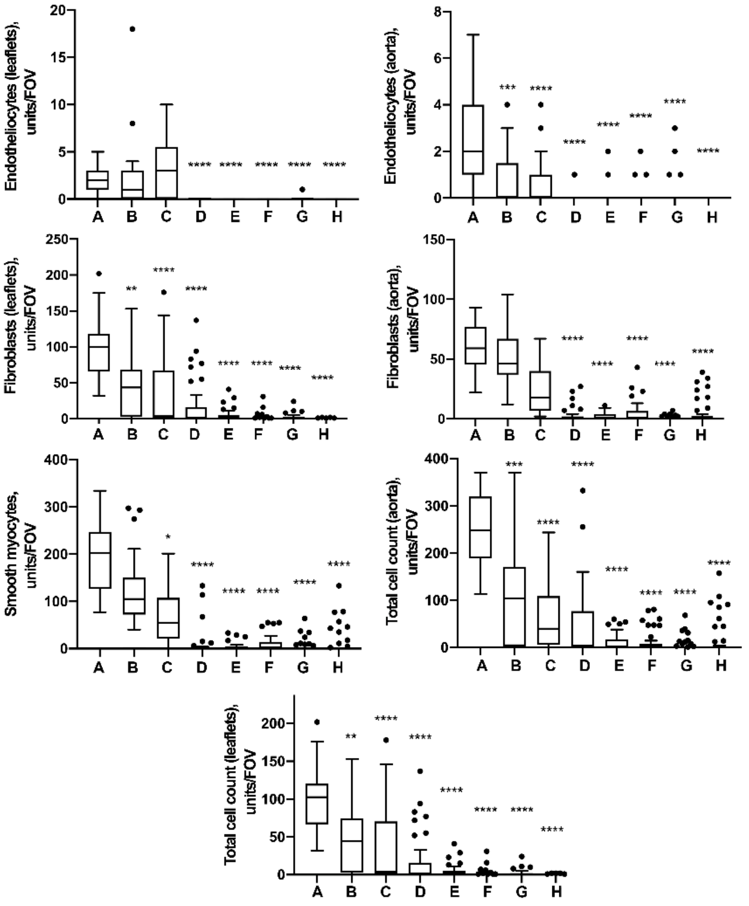

Figure S5. The content of endotheliocytes, fibroblasts and smooth myocytes in aortic valve leaflets and walls of native **(A)** and decellularized ovine aortic roots. Decellularization protocols included scCO$_2$-assisted processing at the pressure of 15 MPa **(B)** and 25 MPa **(C)** with the use of ethanol as a co-solvent; combinations of alkaline treatment with scCO$_2$-assisted processing at the pressure of 10 MPa **(D)** and 15 MPa **(E)**; detergents alone **(F)** or with following scCO$_2$ treatment at the pressure of 15 MPa **(G)** and 25 MPa **(H)**. Differences between the groups were analyzed by Kruskal–Wallis test followed by post-hoc Dunn's test. Middle horizontal line in each box with whiskers (Tukey) represents the median, dots represent extreme points. *p <0.05, **p <0.005, ***p<0.001, ****p <0.0001 vs. native sample **(A)**.

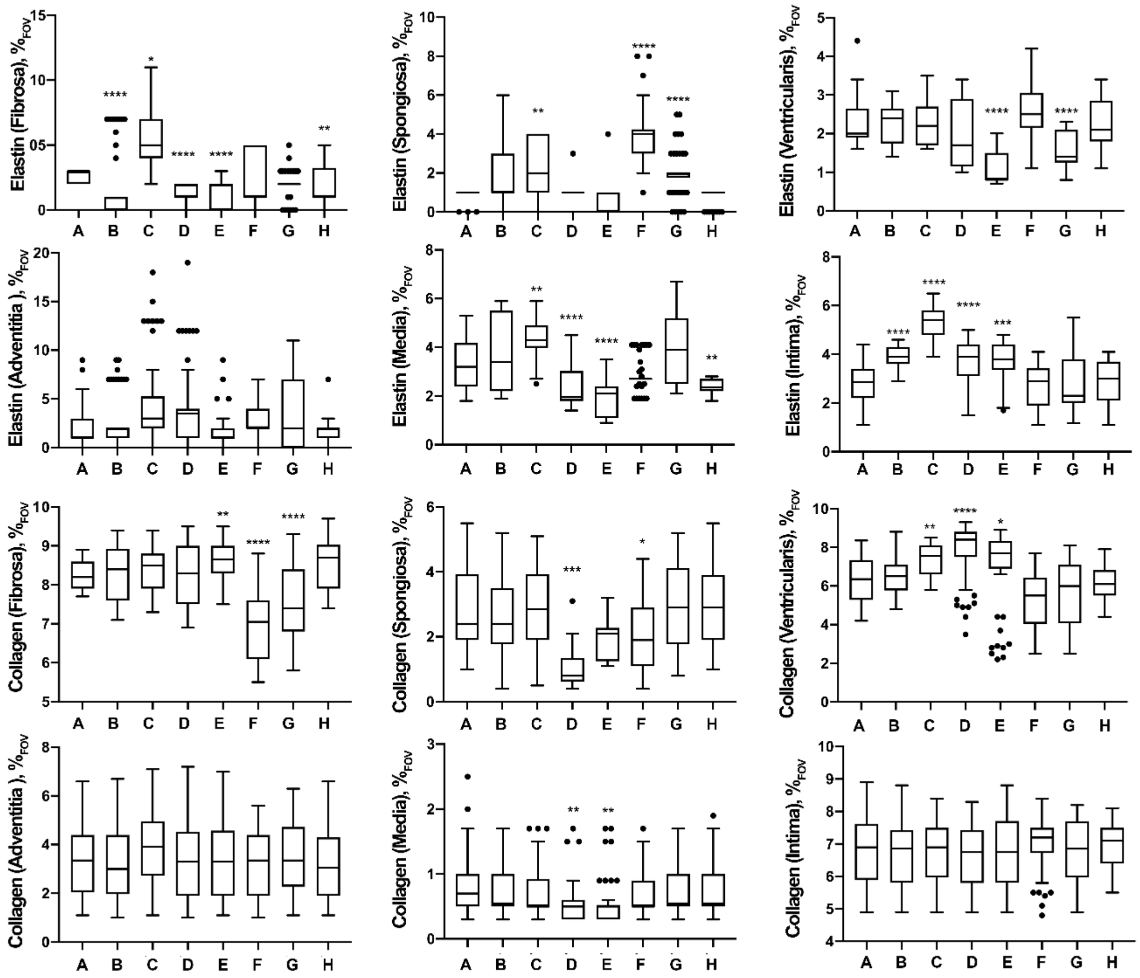

Figure S6. The density of collagen and elastin in aortic valve leaflets and walls of native **(A)** and decellularized ovine aortic roots. Decellularization protocols included scCO2-assisted processing at the pressure of 15 MPa **(B)** and 25 MPa **(C)** with the use of ethanol as a co-solvent; combinations of alkaline treatment with scCO2-assisted processing at the pressure of 10 MPa **(D)** and 15 MPa **(E)**; detergents alone **(F)** or with following scCO2 treatment at the pressure of 15 MPa **(G)** and 25 MPa **(H)**. Differences between the groups were analyzed by one-way Brown-Forsythe ANOVA followed by Dunnett's T3 post-hoc test (for collagen in fibrosa, spongiosa, intima, adventitia and elastin in ventricularis, intima) or Kruskal–Wallis test followed by post-hoc Dunn's test (for collagen in ventricularis, media and elastin in fibrosa, spongiosa, media, adventitia). Middle horizontal line in each box with whiskers (Tukey) represents the median, dots represent extreme points. *p <0.05, **p <0.005, ***p<0.001, ****p <0.0001 vs. native sample **(A)**.

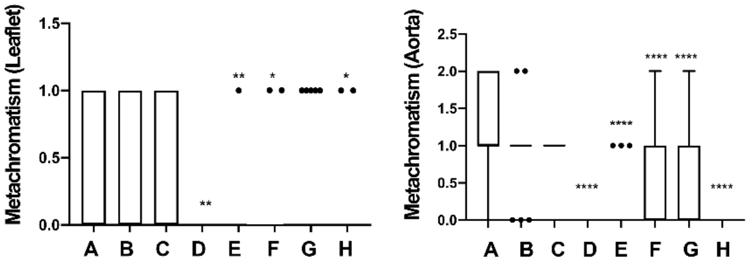

Figure S7. Toluidine blue-assisted metachromatism of aortic valve leaflets and walls of native **(A)** and decellularized ovine aortic roots scored with score 0 – none, score 1 – weak, score 2 - pronounced. Decellularization protocols included scCO2-assisted processing at the pressure of 15 MPa **(B)** and 25 MPa

(C) with the use of ethanol as a co-solvent; combinations of alkaline treatment with $scCO_2$-assisted processing at the pressure of 10 MPa **(D)** and 15 MPa **(E)**; detergents alone **(F)** or with following $scCO_2$ treatment at the pressure of 15 MPa **(G)** and 25 MPa **(H)**. Differences between the groups were analyzed by Kruska–Wallis test followed by post-hoc Dunn's test. Middle horizontal line in each box with whiskers (Tukey) represents the median, dots represent extreme points. *p <0.05, **p <0.005, ***p<0.001, ****p <0.0001 vs. native sample **(A)**.

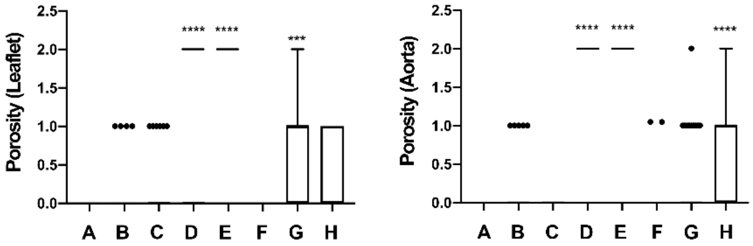

Figure S8. The porosity of aortic valve leaflets and walls of native **(A)** and decellularized ovine aortic roots scored with score 0 – none, score 1 – weak, score 2 - pronounced. Decellularization protocols included $scCO_2$-assisted processing at the pressure of 15 MPa **(B)** and 25 MPa **(C)** with the use of ethanol as a co-solvent; combinations of alkaline treatment with $scCO_2$-assisted processing at the pressure of 10 MPa **(D)** and 15 MPa **(E)**; detergents alone **(F)** or with following $scCO_2$ treatment at the pressure of 15 MPa **(G)** and 25 MPa **(H)**. Differences between the groups were analyzed by Kruskal–Wallis test followed by post-hoc Dunn's test. Middle horizontal line in each box with whiskers (Tukey) represents the median, dots represent extreme points. *p <0.05, **p <0.005, ***p<0.001, ****p <0.0001 vs. native sample (A).

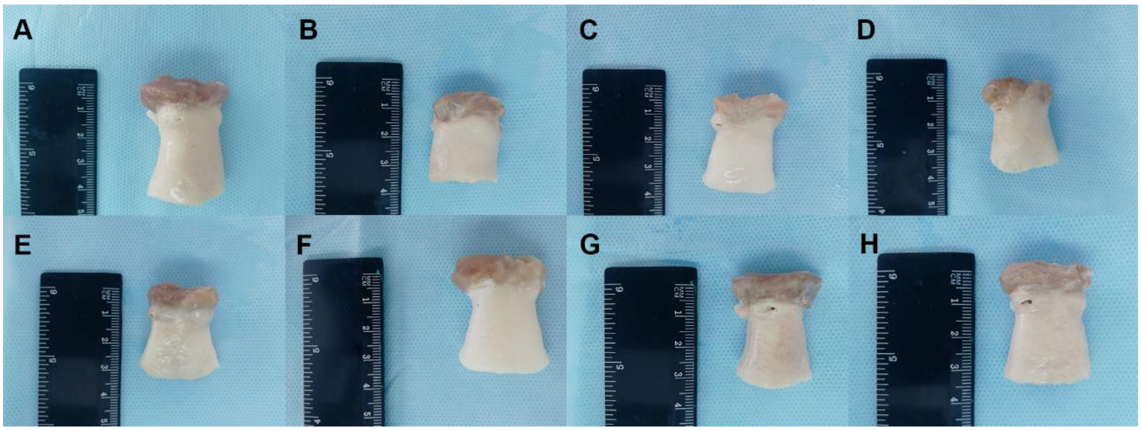

Figure S9. The gross appearance of native **(A)** and decellularized ovine aortic roots. Decellularization protocols included $scCO_2$-assisted processing at the pressure of 15 MPa **(B)** and 25 MPa **(C)** with the use of ethanol as a co-solvent; combinations of alkaline treatment with $scCO_2$-assisted processing at the pressure of 10 MPa **(D)** and 15 MPa **(E)**; detergents alone **(F)** or with following $scCO_2$ treatment at the pressure of 15 MPa **(G)** and 25 MPa **(H)**.

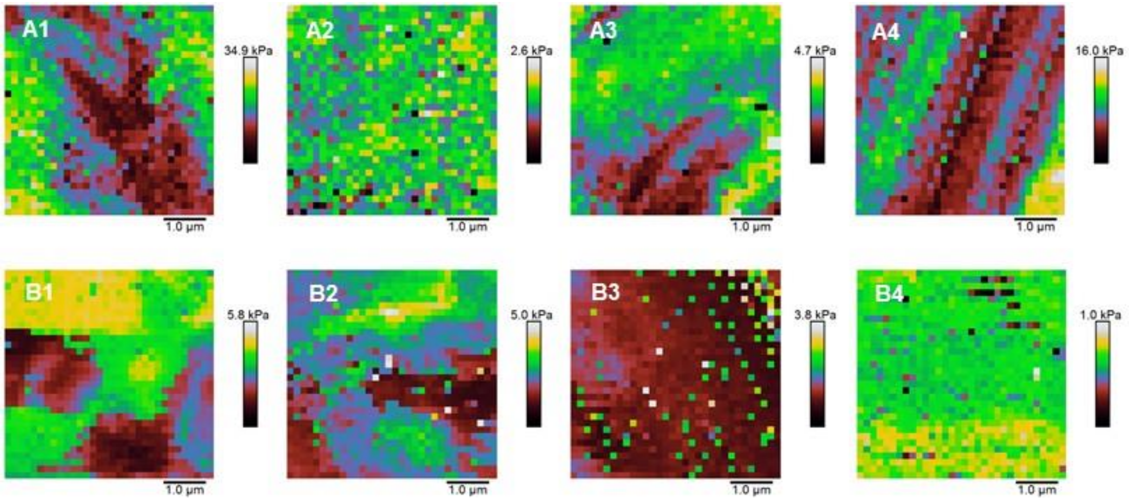

Figure S10. Mapping the local Young's modulus of ovine aortic roots decellularized in a 0.5% SDS/0.5% SD detergent solution **(A1-4)** and after the subsequent processing in $scCO_2$ medium (T = 37 °C, P = 25 MPa, t = 3 h) **(B1-4)** using atomic force microscopy.

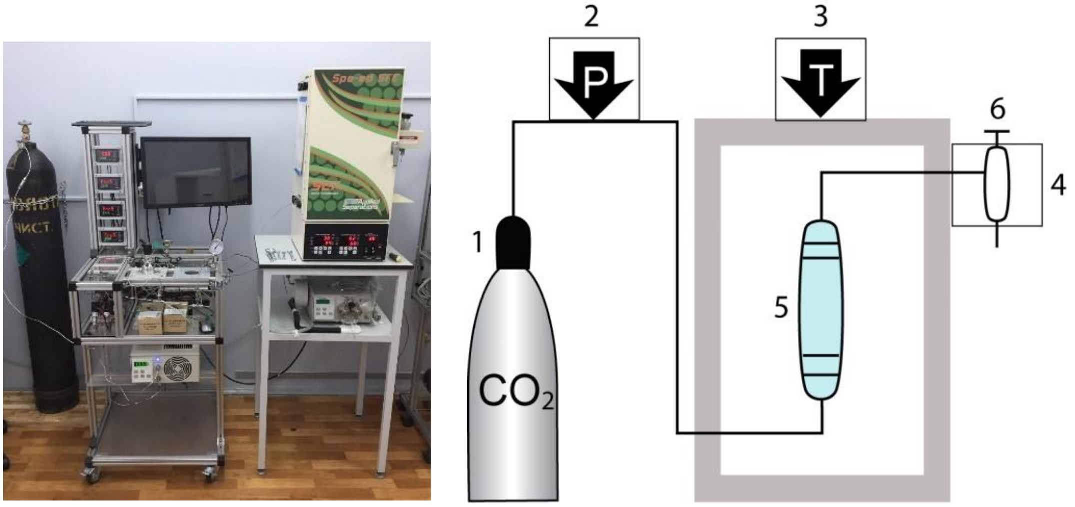

Figure S11. The experimental setup (1 - cylinder with CO_2, 2 - pump, 3 - reactor thermostat, 4 - regulating valve thermostat, 5 - reactor, 6 - fine adjustment valve).

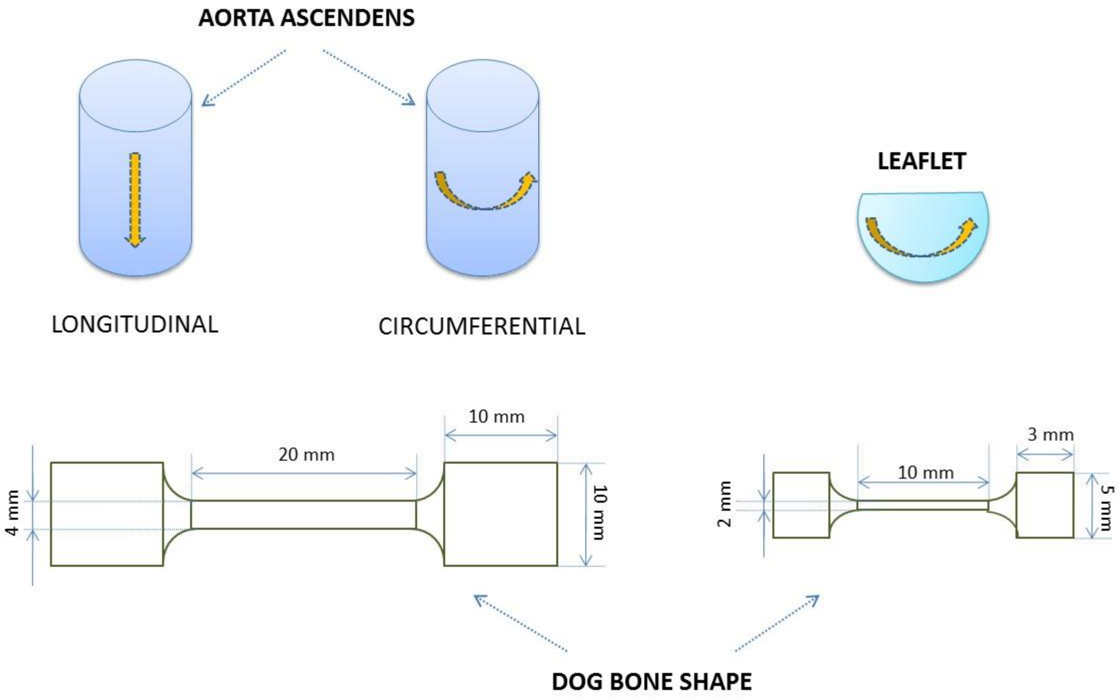

Figure S12. Schematic representation of the sample preparation for uniaxial tensile testing.

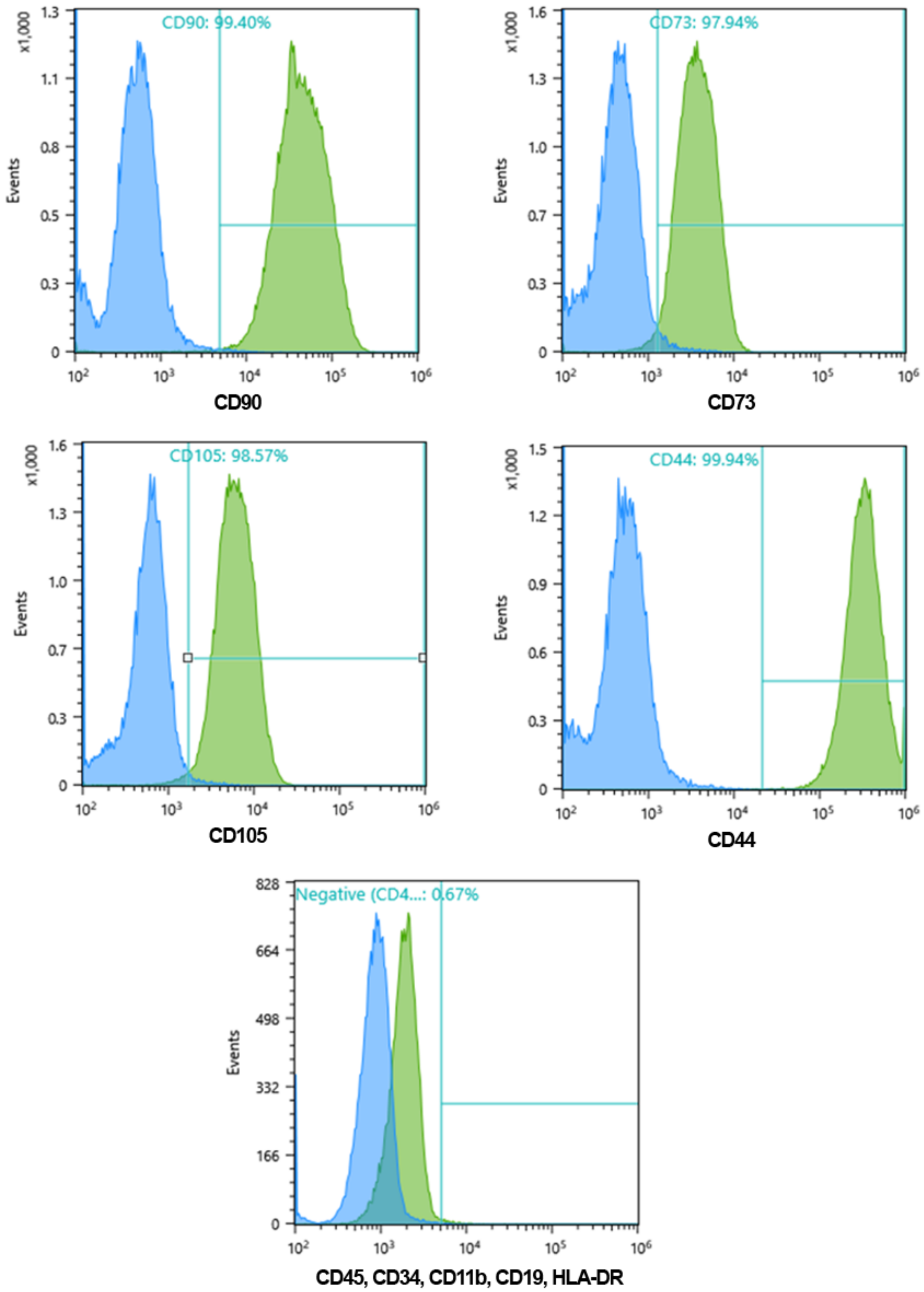

Figure S13. Characterization of the human umbilical cord multipotent mesenchymal stromal cells by flow cytometry. The cells (passage 4) were stained for the specific surface markers as described in Materials and Methods section. Expanded cells demonstrated the expression of CD44, CD105, CD90, CD73 and were negative for CD45, CD34, CD11b, CD19 and HLA-DR (green). Isotype controls conjugated with PE (blue) was run for the comparison.
